# The Knight Alzheimer Research Imaging (KARI) dataset: a comprehensive multimodal resource for exploring aging, preclinical, and symptomatic Alzheimer disease pathology

**DOI:** 10.21203/rs.3.rs-7962593/v1

**Published:** 2025-11-07

**Authors:** David A. Hoagey, Nicole S. McKay, Nelly Joseph-Mathurin, Shaney Flores, Stephanie Doering, Sarah J. Keefe, Russ C. Hornbeck, Thomas H. Smith, Jalen Scott, Gengsheng Chen, Parinaz Massoumzadeh, Pamela J. LaMontagne, Qing Wang, Jason Hassenstab, Maria Rosana Ponisio, Andrea Denny, Joyce E. Balls-Berry, B. Joy Snider, Susan L. Stark, Chengjie Xiong, Suzanne E. Schindler, Richard J. Perrin, Joshua S. Shimony, Manu S. Goyal, Andrei G. Vlassenko, Marcus E. Raichle, John C. Morris, Cyrus A. Raji, Brian A. Gordon, Tammie L. S. Benzinger

**Affiliations:** 1 Department of Radiology, Washington University School of Medicine, Saint Louis, MO, United States.; 2 Department of Neurology, Washington University School of Medicine, Saint Louis, MO, United States; 3 Division of Biostatistics, Washington University School of Medicine, Saint Louis, MO, United States; 4 Department of Psychological & Brain Sciences, Washington University in St. Louis, Saint Louis, MO, United States; 5 Department of Pathology and Immunology, Washington University School of Medicine, Saint Louis, MO, United States; 6 Program in Occupational Therapy, Washington University School of Medicine, Saint Louis, MO, United States

## Abstract

Alzheimer disease (AD) remains a significant global public health challenge, requiring robust multimodal datasets to elucidate its prolonged preclinical phase, improve early detection, advance understanding of disease trajectories, and guide intervention strategies. To address this, the Charles F. and Joanne Knight Alzheimer Disease Research Center (Knight ADRC) at Washington University in St. Louis established the Knight Alzheimer Research Imaging (KARI) dataset. This paper characterizes this dataset emphasizing its phenotypical depth and longitudinal scope, detailing comprehensive multimodal neuroimaging from 1,645 participants (aged 42–97) across 6,217 acquisitions. Spanning the AD spectrum from healthy aging to symptomatic disease, the cohort undergoes extensive longitudinal imaging using structural and functional magnetic resonance imaging (MRI) alongside positron emission tomography (PET) tracers for amyloid and tau pathology. This is complemented by rich clinical, cognitive, genetic, and biomarker data. In addition to raw imaging, the dataset provides quality-controlled processed outputs, including anatomical segmentations and biomarker quantification. By making this data accessible to researchers, the Knight ADRC aims to accelerate discoveries in pathophysiology, biomarker identification, and therapeutic development.

## Introduction

### Overview of ADRD datasets

Alzheimer disease (AD) affects more than 57 million people worldwide, and this number continues to rise as populations age (Nichols et al., 2022). The growing prevalence of AD highlights an urgent need to better understand its earliest stages, when intervention may have the greatest impact. A major challenge is characterizing the long preclinical phase of sporadic AD, which can extend for decades before cognitive symptoms emerge (Li et al., 2024; Villemagne et al., 2013). Intervening during this period offers the greatest potential to alter disease trajectories. Elucidating this preclinical phase depends on longitudinal datasets that capture subtle biological and cognitive changes over time, combined with multimodal imaging to map the complex, often heterogenous pathology of asymptomatic individuals. Mahor multi-site efforts such as the Alzheimer’s Disease Neuroimaging Initiative (ADNI)(Weiner et al., 2025) and the Dominantly Inherited Alzheimer Network (DIAN)(Bateman et al., 2012; McKay et al., 2023) have provided important biomarker insights. However, ADNI initially emphasized symptomatic recruitment, and DIAN focuses on rare familial variants. Comprehensive, open-access resources that focus on individuals at risk for sporadic, late-onset AD are therefore essential to characterize the presymptomatic and earliest symptomatic stages in representative populations.

### Composition of KARI dataset

The Charles F. and Joanne Knight Alzheimer Disease Research Center (Knight ADRC) at Washington University in St. Louis School of Medicine is dedicated to advancing our understanding of AD through its longitudinal research program that includes the Knight Alzheimer Research Imaging (KARI) dataset. KARI addresses the gap in available datasets by focusing on deep phenotyping across the AD spectrum, with strong representation of preclinical participants. The dataset primarily includes participants from two long-running, complementary longitudinal studies: the Healthy Aging and Senile Dementia (HASD) program and the Adult Children Study (ACS). HASD enrolls cognitively normal older adults to characterize the transition to symptomatic disease, whereas ACS follows a younger at-risk cohort to capture the early biomarker changes in mid-life. Crucially, both studies utilize a shared, uniform assessment protocol that enables harmonized analyses across the disease continuum. These data are collected, curated, and shared through collaborative efforts across Washington University’s Kight ADRC’s research cores, providing a framework to investigate how biological and cognitive markers evolve throughout AD’s prolonged preclinical course.

### Novelty of KARI dataset

The KARI dataset’s scale, longitudinal depth, and accessibility distinguish it from other AD resources. As of September 2024, data includes values from over 1,600 participants and more than 6,200 neuroimaging sessions, with repeated measures from over 800 individuals spanning the older adult lifespan, some followed for more than 15 years. Available modalities encompass high-resolution structural, diffusion, and functional MRI, amyloid and tau-PET, clinical and cognitive assessments, genetic data, neuorpathology, and fluid biomarkers. Standardized acquisition and processing pipelines maintain data quality and consistency over time. This integrated framework supports investigations into the temporal evolution and interplay of AD pathologies, enabling a more holistic understanding of disease mechanisms. The Knight ADRC provides qualified investigators with access to both raw and processed data, supporting open and reproducible analyses.

### Overview and aims

In this report, we describe the KARI dataset, detailing its longitudinal, multimodal neuroimaging, and preclinical characteristics, to illustrate its potential for advancing AD research. We summarize the available acquisitions across each neuroimaging modality, describe the composition of the dataset in terms of participant numbers and longitudinal follow-up, and summarize the key demographic and clinical characteristics of the study cohort. Furthermore, we will examine how imaging biomarkers relate to clinical status, indexed by the Clinical Dementia Rating^®^ (CDR^®^) (Morris, 1993). This detailed overview provides researchers with the context needed to access and utilize this expansive resource for their own investigations of AD and related dementias.

## Results

### Data Characterization

**Figure 1:**
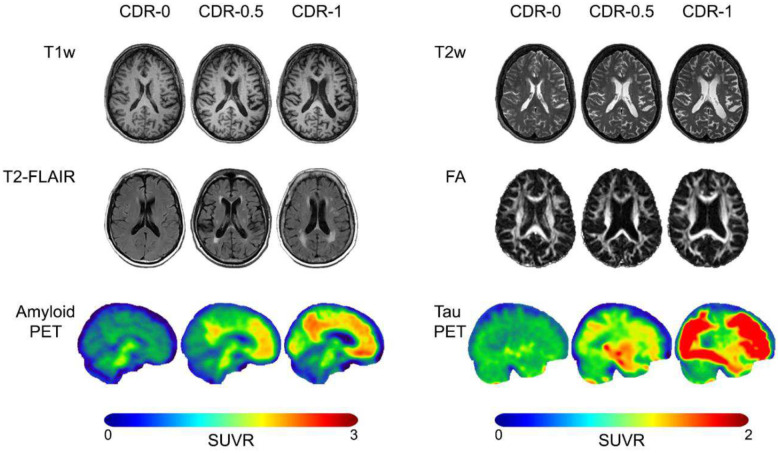
Example brain images from select acquisitions including T1-weighted (T1w) Magnetization Prepared Rapid Gradient Echo (MPRAGE), T2-weighted (T2w) Sampling Perfection with Application optimized Contrasts using different flip angle Evolutions (SPACE), T2 Fluid-Attenuated Inversion Recovery (T2-FLAIR), Diffusion Fractional Anisotropy (FA), Amyloid-PET using ^11^C-Pittsburgh Compound B, and Tau-PET using ^18^F-Flortaucipir. Columns demonstrate examples from across clinical status according to the Clinical Dementia Rating (CDR). T1w and T2w structural images are from the same participant followed longitudinally. T2-FLAIR, FA, Amyloid-PET, and Tau-PET examples are pulled from different participants to capture each CDR rating. All images are registered to MNI space for anatomical comparability.

**Figure 2:**
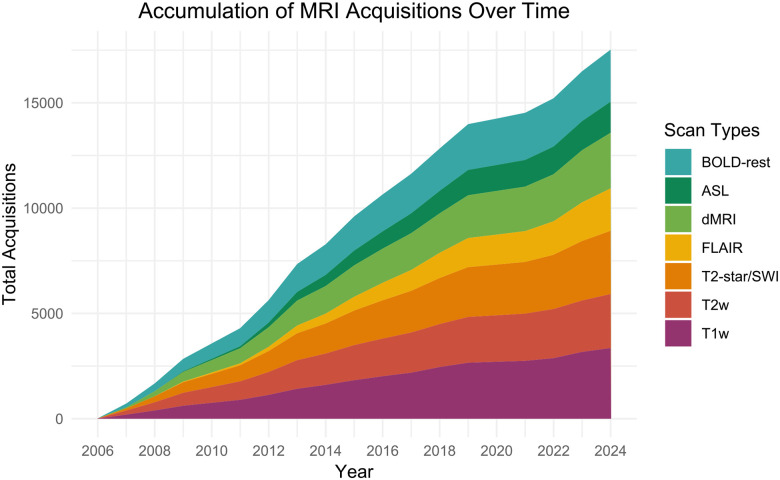
Stacked area plot illustrating the cumulative number of MRI acquisitions, grouped by scan type, over time. Acquisitions include T1-weighted (T1w), T2-weighted (T2w), T2-star, Gradient-Recalled Echo (GRE), Susceptibility-Weighted Imaging (SWI), diffusion-weighted (dMRI), T2 Fluid-Attenuated Inversion Recovery (T2-FLAIR), Arterial Spin Labeling (ASL), and resting state Blood Oxygenation Level Dependent (BOLD-rest). T2-star, GRE, and SWI data were acquired with varying protocols and are grouped under T2-star/SWI to reflect harmonization efforts for microhemorrhage detection.

Data analyzed here was collected prior to September 30^th^, 2024, and released November 30th, 2024 as part of data freeze 25 (DF25). In total, 3,385 separate 3T-MRI sessions were conducted across 1,571 unique individuals with 801 of these individuals having longitudinal follow-up sessions, many with more than two timepoints of data, across an average longitudinal lag of 3.31 years (SD = 1.71; table 1). During these sessions, a comprehensive suite of MRI sequences was acquired, including structural, functional, and diffusion-weighted scans (see Online Methods for a full list of protocols). Example slices from selected modalities are shown in figure 1. Acquisition counts for each protocol are included in Figure 2.

**Figure 3:**
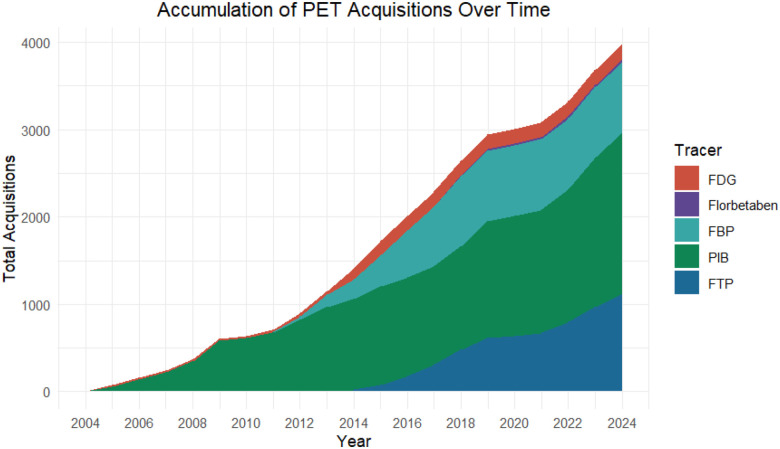
Stacked area plot illustrating the cumulative number of PET acquisitions, grouped by tracer type, over time. Acquisitions include ^18^F-Fluorodeoxyglucose (FDG), ^18^F-Flortaucipir (FTP), ^18^F-Florbetaben (FBB), ^18^F-Florbetapir (FBP), ^11^C-Pittsburgh Compound B (PiB).

PET imaging was conducted using multiple tracers to assess amyloid (^11^C-Pittsburgh Compound B (PiB), ^18^F-Florbetapir (FBP), ^18^F-Florbetaben (FBB)), and tau pathology (^18^F-Flortaucipir (FTP)). 2,684 separate amyloid-PET sessions were conducted across 1,370 unique individuals, with 632 of these individuals having longitudinal follow-up sessions separated by an average lag of 3.38 years (SD = 1.56). Each amyloid tracer was employed to varying degrees, with a primary focus on collecting PiB for current and future acquisitions, totaling 1,848 sessions across 1,108 unique individuals. FBP was acquired in 810 sessions across 660 unique participants, while FBB was acquired in 26 sessions across 17 participants. 1,113 separate tau-PET sessions were conducted, all using the FTP tracer, across 826 unique individuals. 228 of these individuals have longitudinal follow-up sessions separated by an average lag of 3.87 years (SD = 1.55). A detailed breakdown of the study counts, longitudinal breakdown, and an illustration of acquisitions for each PET tracer can be found in table 2 and figure 3.

**Figure 4:**
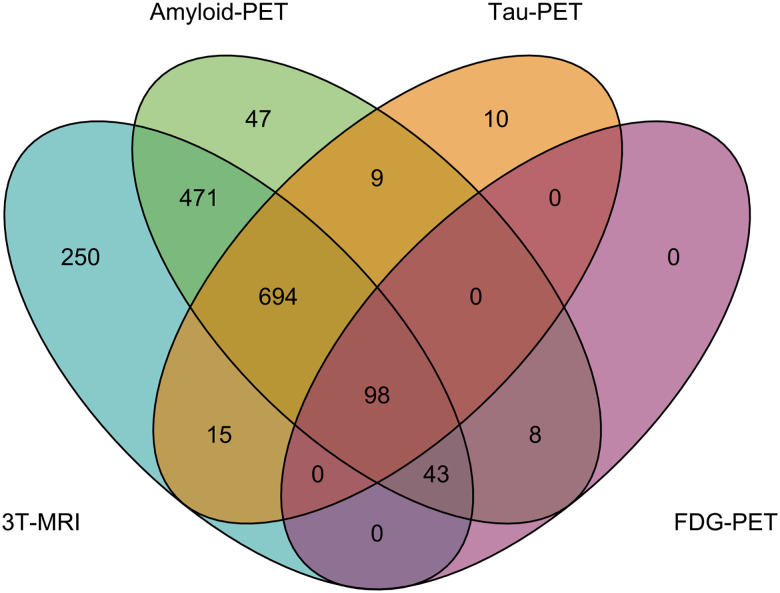
Venn diagram illustrating the number of unique individuals with 3T-MRI, amyloid-PET, tau-PET, and FDG-PET sessions undergone by participants included in data freeze 25. Intersections highlight the overlap for those with data for multiple different types of neuroimaging sessions.

Across each of our primary neuroimaging modalities, 792 individuals have data encompassing all three primary imaging modalities, including at least one 3T-MRI, one amyloid-PET, and one tau-PET session. An additional 514 individuals have completed at least one 3T-MRI and one amyloid-PET session (figure 4).

### Clinical

**Figure 5:**
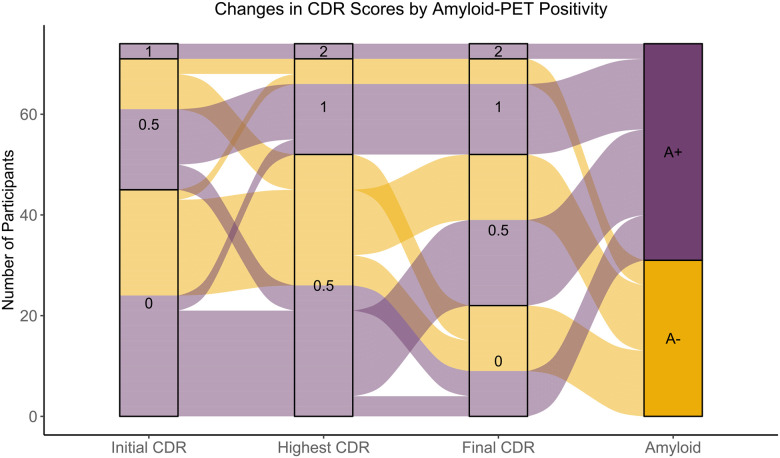
Alluvial plot depicting the Clinical Dementia Rating (CDR) converters according to their initial CDR, highest CDR received, and final (or most recent) CDR score, colored according to their amyloid status defined by an amyloid-PET Centiloid cutoff of 18.42 (see GMM methods). Note that this plot includes the 76 participants who either increased, decreased, or fluctuated in CDR scores, not those who maintained a single CDR or those who only had one cross-sectional assessment (available in table 3).

Cross-sectional and longitudinal characterization of CDR scores are presented in table 3 for the 1643 participants with a neuroimaging session acquired within 2 years of clinical assessment. Most participants (1,479) showed no change in CDR score across the study, including 347 participants who only received one CDR assessment, and 1132 who remained stable longitudinally. Among the remaining 164 participants, 114 had one or more higher scores after baseline, 25 had one or more lower scores after baseline, and 25 fluctuated in their longitudinal CDR score (table 3). The highest CDR score for each participant was as follows: 1,120 participants had a CDR score of 0, 362 had a CDR score of 0.5, 150 had a CDR score of 1, and 11 had a CDR score of 2. This demonstrates the preclinical focus of the dataset with a much greater number of unimpaired individuals. Figure 5 illustrates changes in CDR across the study by initial, maximum, and most recent CDR score, separated by amyloid status for the participants who had undergone amyloid-PET imaging.

### Demographics

**Figure 6:**
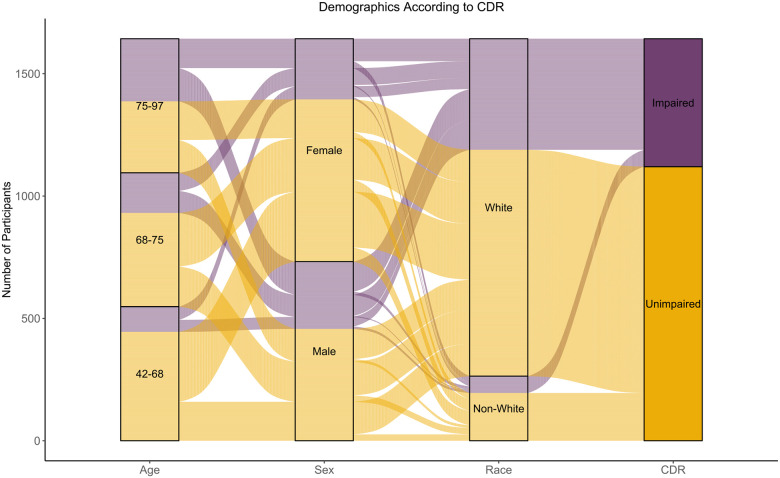
Alluvial plot illustrating the relationship between demographic factors of age, sex, race, and clinical status on the 1643 participants who have at least one neuroimaging session (MRI, amyloid-PET, tau-PET, or FDG-PET) and a Clinical Dementia Rating (CDR) assessment within 2 years. CDR values are the highest value recorded across study participation. Unimpaired – CDR 0; Impaired - CDR >0.5.

Demographic data are acquired from all participants to phenotype our sample along multiple domains. These data include self-reported sex (as either “man” or “woman”), education in years, date of birth, and race, recorded during a participant’s first visit. Socioeconomic status was recorded according to the Hollingshead index (Hollingshead, 2011), while handedness was determined according to a handedness task (Kimura & Vanderwolf, 1970). To summarize demographic data and determine group effects, we analyzed the subset of participants who obtained at least one neuroimaging session with a corresponding CDR assessment obtained within two years. The resulting 1,643 participants in the Knight ADRC cohort have a mean age of 70.00 years (SD = 9.26) and range from 42 to 97 years of age at baseline. Those enrolling with CDR > 0 (impairment rated as very-mild to severe) are significantly older (Mean= 74.58) than those with CDR = 0 (unimpaired) tested using a Welch’s t-test to account for unequal variances (Mean = 68.40; t(877.60) = 13.47, *p* < .0001, Cohen’s d = 0.70). We then analyzed demographics according to each participant’s highest CDR throughout study participation. The cohort is highly educated, with an average of 15.86 years of education. Impaired individuals attained fewer years of education (M = 15.23) than unimpaired individuals (M = 16.15), a significant difference (t(883.32) = −6.31, *p* < .0001, Cohen’s d = −0.35). Women comprise 55.53% of the sample (n = 911), with a higher proportion in the unimpaired group (n = 663, 59.20%) compared to the impaired group (n = 248, 47.42%), a statistically significant difference (χ^2^(1,1643) = 19.55, *p* < .0001, φ = 0.11). The cohort is predominantly right-handed (n = 1422, 86.55%), with no significant differences between impaired and unimpaired groups (χ^2^(2,1643) = 0.27, *p* = .8743, Cramer’s V = .01). Most participants are White (n = 1379, 83.93%), while 245 (14.91%) identify as Black, a proportion that meets or exceeds population estimates for the St. Louis metropolitan area. An additional 10 (0.61%) identify as Asian, and 9 (0.55%) identified themselves as either “other” or “multiple races”. No significant group differences were found between race and CDR status (χ^2^(3,1643) = 5.89, *p* = .1173, Cramer’s V = .06). Apolipoprotein E (*APOE*) ε4 carriers—those with at least one ε4 allele—make up 42.06% of the cohort (n = 691). The impaired group has a significantly higher proportion of ε4 carriers (56.02%) than the unimpaired group (35.53%; χ^2^ (1,1643) = 60.57, *p* < .0001, φ = .19; Table 4). Graphical depiction of the participant demographic breakdown according to age, sex, race, and CDR status is presented in figure 6.

### Cognition

**Figure 7:**
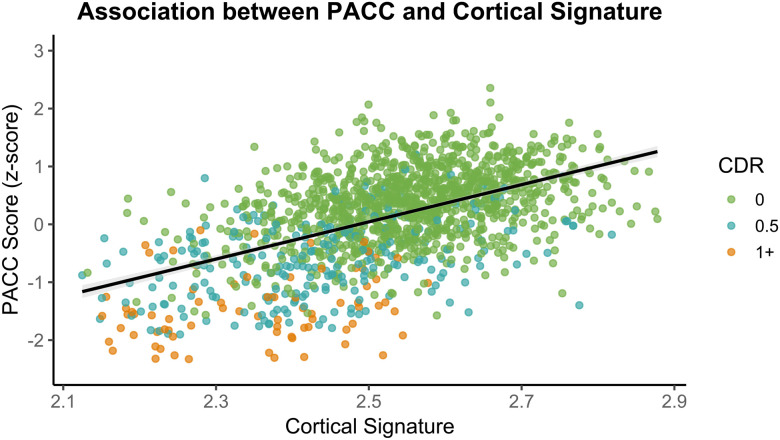
Scatterplot depicting the association between cortical signature thickness (x-axis) and Preclinical AD Cognitive Composite (PACC) scores (y-axis) across individuals stratified by Clinical Dementia Rating (CDR) status (0 = cognitively normal, 0.5 = very mild impairment, 1+ = mild-to-moderate impairment). Each point represents a single observation, colored by CDR group. The overlaid black regression line represents the linear association controlling for age, education, sex, and APOE ε4 status. Shaded gray band denotes the 95% confidence interval around the fitted regression line. Overall, greater cortical signature thickness was associated with better cognitive performance, as measured by the PACC, independent of clinical severity group.

**Figure 8:**
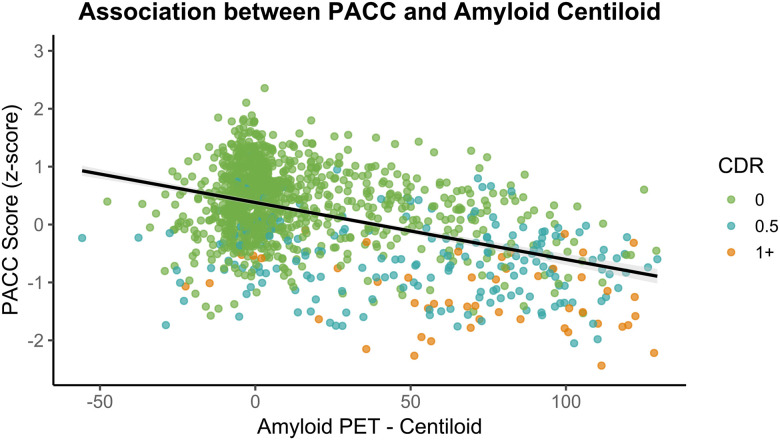
Scatterplot depicting the association between amyloid Centiloid (x-axis) and Preclinical AD Cognitive Composite (PACC) scores (y-axis) across individuals stratified by Clinical Dementia Rating (CDR) status (0 = cognitively normal, 0.5 = very mild impairment, 1+ = mild-to-moderate impairment). Each point represents a single observation, colored by CDR group. The overlaid black regression line represents the linear association controlling for age, education, sex, and APOE ε4 status. Shaded gray band denotes the 95% confidence interval around the fitted regression line. Overall, increased amyloid deposition was associated with worse cognitive performance, as measured by the PACC, independent of clinical severity group.

**Figure 9:**
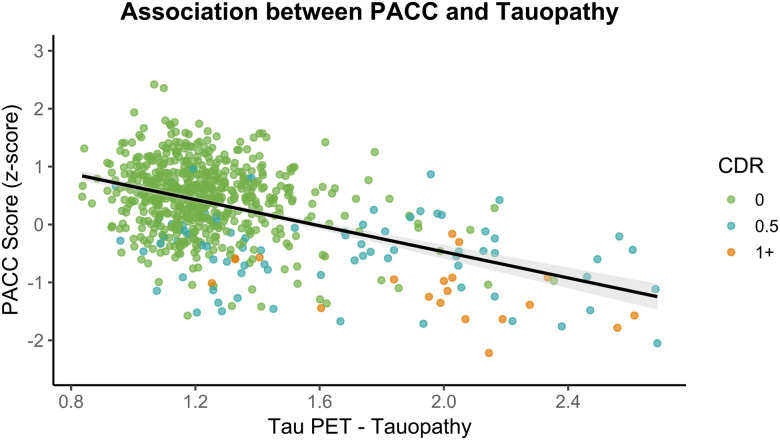
Scatterplot shows the association between tauopathy (x-axis) and Preclinical AD Cognitive Composite (PACC) scores (y-axis) across individuals stratified by Clinical Dementia Rating (CDR) status (0 = cognitively normal, 0.5 = very mild impairment, 1+ = mild-to-moderate impairment). Each point represents a single observation, colored by CDR group. The overlaid black regression line represents the linear association controlling for age, education, sex, and APOE ε4 status. Shaded gray band denotes the 95% confidence interval around the fitted regression line. Overall, greater tau accumulation was associated with worse cognitive performance, as measured by the PACC, independent of clinical severity group.

Cognitive performance was summarized using the Knight Preclinical AD Cognitive Composite (Knight PACC), as detailed in McKay et al., 2024 and in the Online Methods. To examine the association between AD biomarkers and cognitive performance, we conducted partial Pearson correlations between the PACC cognitive composite score and three imaging-based summary metrics representing cortical thickness or signature, amyloid burden as measured with the Centiloid scale, and tau burden or Tauopathy, controlling for age, years of education, sex, and APOE ε4 carrier status (details of each metric can be found in the Online Methods). Analyses were conducted on participants with both imaging and cognitive data acquired within two years. Among the 1,467 participants with MRI data, the average PACC score was 0.13 (SE = 0.02). A significant positive partial correlation was observed between cortical signature and PACC score, r(1461) = .36, *p* < .0001, 95% CI [0.32, 0.41], indicating that lower cortical thickness in AD-signature regions was moderately associated with lower cognitive performance (figure 7). For amyloid-PET, analysis included 1,293 participants with a mean PACC score of 0.20 (SE = 0.02). A significant negative partial correlation was observed between Centiloid values and PACC score, r(1287) = −.33, *p* < .0001, 95% CI [−0.38, −0.29], suggesting that greater amyloid burden was associated with lower cognitive performance (figure 8). For tau-PET, 684 participants were included, with a mean PACC score of 0.33 (SE = 0.03). A significant negative partial correlation was also found between Tauopathy and PACC score, r(678) = −.40, *p* < .0001, 95% CI [−0.47, −0.34], indicating that higher tau burden was associated with worse cognitive performance (figure 9). Together, these findings demonstrate robust and directionally consistent relationships between AD biomarker load and cognitive functioning.

### Imaging Variable

#### Neurodegeneration

**Figure 10:**
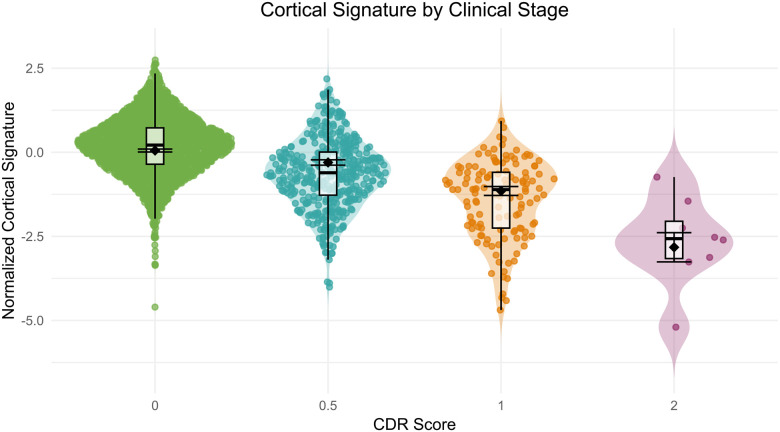
Violin plot of MRI-based cortical signature grouped by Clinical Dementia Rating (CDR) for the 3025 3T-MRI sessions with an accompanying CDR assessment within 2 years. Cortical signature values are z-scored and larger values indicate greater cortical thickness. Individual data points are plotted and randomly jittered to aid visualization. Group means accounting for age are plotted as horizontal dashed lines illustrating increased cortical thinning with worsening CDR score as expected.

To assess neurodegeneration, we used cortical signature, an established composite measure of cortical thickness in regions sensitive to early AD-related atrophy (see Online Methods for details). This measure is calculated for each data freeze to serve as a key marker of neurodegenerative severity. Modeling cortical signature by CDR status, there are significant group differences in cortical signature between CDR = 0 (M = 2.55, SD = 0.12) and CDR > 0 (M = 2.41, SD = .15), t(3023) = −23.84, *p* < .0001, 95% CI [−0.15, −0.13]. Figure 10 illustrates this decrease in cortical signature with increasing CDR across the 3025 MRI sessions with which a CDR assessment was obtained within 2 years, highlighting the differences along the AD continuum.

Similarly, there are age-related differences in cortical signature across CDR levels. A linear mixed-effects model was fitted with age (centered), CDR, and their interaction as fixed effects, and random intercepts for participants to account for repeated measures. Chronological age was used as the longitudinal time scale. Results revealed a significant main effect of age (β = −0.061, SE = 0.0018, t(2976) = −34.68, *p* < .0001), indicating that cortical signature decreases with increasing age. There were also significant main effects of CDR, such that compared to CDR 0, cortical signature was significantly lower for individuals with CDR 0.5 (β = −0.356, SE = 0.041, t(2948) = −8.59, *p* < .0001), CDR 1 (β = −1.203, SE = 0.070, t(2838) = −17.25, *p* < .0001), and CDR 2 (β = −2.878, SE = 0.222, t(2815) = −12.94, *p* < .0001). The omnibus age by CDR interaction was significant, F(3, 2740) = 6.40, p = .0003. Importantly, follow-up contrasts revealed a significant interaction for CDR 1 (β = 0.028, SE = 0.0077, t(2702) = 3.66, *p* = .0003), suggesting that the negative effect of age on cortical signature was attenuated in individuals with CDR 1. Pairwise comparisons of estimated marginal means revealed significant differences in cortical signature across all CDR levels (*p* < .0001, Tukey adjusted). Specifically, cortical signature (z-scored) decreased monotonically across increasing CDR, with estimated marginal means as follows: CDR 0 = 0.053, CDR 0.5 = −0.303, CDR 1 = −1.150, and CDR 2 = −2.824. Model adjusted error bars are included in figure 10 to visualize the impact of age against the unadjusted boxplot.

#### Amyloid-PET

**Figure 11:**
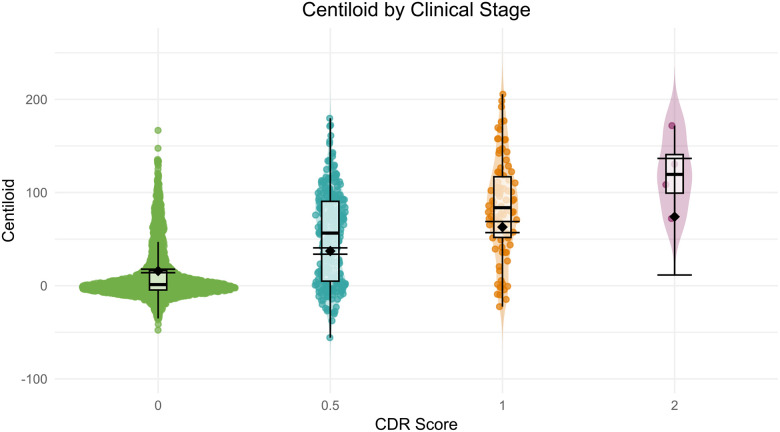
Violin plot of amyloid-PET Centiloid grouped by Clinical Dementia Rating (CDR) for the 2559 3T-MRI sessions with an accompanying CDR assessment within 2 years. Individual data points are plotted and randomly jittered to aid visualization. Group means accounting for age are plotted as horizontal dashed lines illustrating increases in Centiloid with worsening CDR score as expected.

Centiloid composites were used to quantify amyloid-PET accumulation across tracers (for details see Online Methods). Modeling Centiloid by CDR status, there are significant group differences in Centiloid values between CDR = 0 (M = 11.52, SD = 28.36) and CDR > 0 (M = 60.72, SD = 52.78), t(2557) = 26.96, *p* < .0001, 95% CI [45.62, 52.79]. Figure 11 illustrates this increase in Centiloid with increasing CDR across the 2559 amyloid-PET sessions for which a CDR assessment was obtained within 2 years, highlighting the differences along the AD continuum.

Age-related differences in Centiloid were modeled across CDR score using the same mixed-effects framework to account for the longitudinal structure of the data. Results revealed a significant main effect of age (β = 0.94, SE = 0.07, t(2174) = 13.97, *p* < .0001), indicating that Centiloid levels increased with increasing age. There was also a significant main effect of CDR, with individuals at CDR 0.5 (β = 21.39, SE = 1.70, t(2104) = 12.55, *p* < .0001) and CDR 1 (β = 47.10, SE = 3.08, t(2543) = 15.29, *p* < .0001) showing significantly higher Centiloid levels relative to those at CDR 0. Although the effect for CDR 2 was not statistically significant at the conventional alpha level (β = 58.20, SE = 31.90, t(1692) = 1.82, *p* = .0683), the large effect size and wide confidence interval suggest potential variability due to smaller sample size. The omnibus age by CDR interaction was significant, F(3, 2065) = 7.72, p < .0001. Follow-up contrasts indicated a significant interaction for CDR 1 (β = −1.63, SE = 0.34, t(2550) = −4.80, *p* < .0001), suggesting that the rate of Centiloid accumulation with age was attenuated relative to those with CDR 0. No significant age interactions were found for CDR 0.5 or CDR 2. Estimated marginal means further confirmed increasing Centiloid burden with advancing CDR stage: CDR 0 = 15.9 (0.96), CDR 0.5 = 37.3 (1.74), CDR 1 = 63.0 (3.03), and CDR 2 = 74.1 (31.90). Pairwise comparisons (Tukey-adjusted) revealed significant differences among several CDR groups (*p* < .0001), except for comparisons involving CDR 2, which were non-significant, likely reflecting reduced statistical power afforded by this smaller subgroup. Model adjusted error bars are included in figure 11 to visualize the impact of age against the unadjusted boxplot.

#### Tau-PET

**Figure 12:**
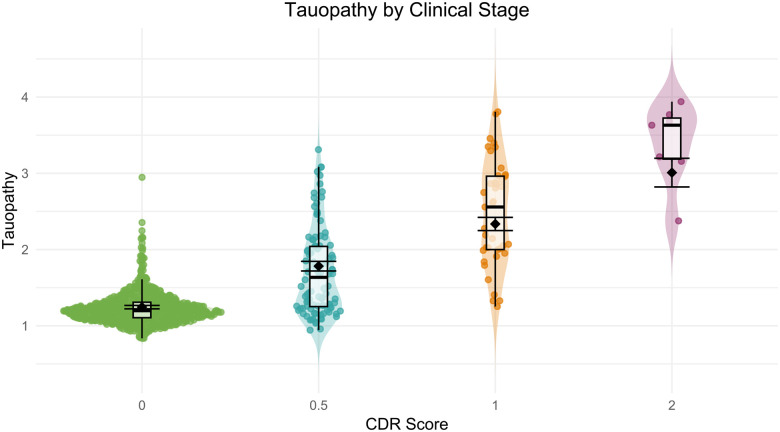
Violin plot of tau-PET tauopathy grouped by Clinical Dementia Rating (CDR) for the 931 tau-PET sessions with an accompanying CDR assessment within 2 years. Tauopathy values are partial volume corrected SUVR. Individual data points are plotted and randomly jittered to aid visualization. Group means accounting for age are plotted as horizontal dashed lines illustrating increases in Tauopathy with worsening CDR score as expected.

To quantify overall tau burden, we used the “tauopathy” summary measure, a composite SUVR score derived from regions known to show early tau accumulation (see Online Methods). Modeling by CDR status, there are significant group differences in tauopathy between CDR=0 (M=1.23, SD=0.21) and CDR > 0 (M = 2.00, SD=0.75), t(929) = 24.23, *p* < .0001, 95% CI [0.71,0.83]. Figure 12 illustrates this increase in tauopathy with increasing CDR across the 931 tau-PET sessions for which a CDR assessment was obtained within 2 years, highlighting the differences along the AD continuum.

Age-related differences in tauopathy were modeled across CDR score using the same mixed-effects framework to account for the longitudinal nature of the data. Results revealed a significant main effect of age (β = 0.009, SE = 0.001, t(889) = 7.90, *p* < .0001), indicating that, overall, tauopathy increased with increasing age. There were also significant effects of CDR 0.5 (β = 0.54, SE = 0.034, t(913) = 15.95, *p* < .0001), CDR 1 (β = 1.09, SE = 0.045, t(907) = 24.01, *p* < .0001), and CDR 2 (β = 1.76, SE = 0.096, t(439) = 18.35, *p* < .0001), suggesting higher tauopathy levels at all impairment stages compared to the unimpaired group. The omnibus age by CDR interaction was significant, F(3,664) = 48.85, p < .0001. Follow-up contrasts showed significant interactions for CDR 0.5 (β = −0.028, SE = 0.004, t(751) = −7.73, *p* < .0001), CDR 1 (β = −0.051, SE = 0.005, t(864) = −10.14, *p* < .0001), and CDR 2 (β = −0.039, SE = 0.009, t(417) = −4.35, *p* < .0001), indicating that the age effect was attenuated in all impaired groups. Estimated marginal means revealed significant differences in tauopathy across CDR stages (*p* < .0001 for all pairwise comparisons, Tukey adjusted), with the following estimated means (SE): CDR 0 = 1.25 (0.01), CDR 0.5 = 1.78 (0.03), CDR 1 = 2.34 (0.04), and CDR 2 = 3.01 (0.10). Post-hoc comparisons confirmed significant differences between all pairs of CDR groups (*p* < .0001). Model adjusted error bars are included in figure 12 to visualize the impact of age against the unadjusted boxplot.

**Figure 13:**
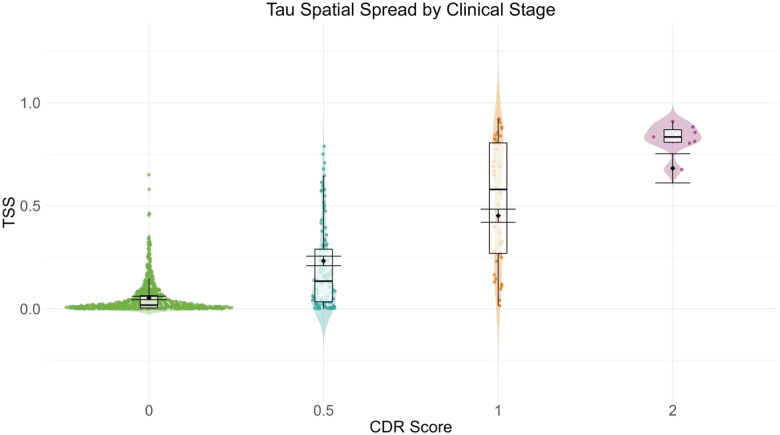
Violin plot of tau spatial spread (TSS) grouped by Clinical Dementia Rating (CDR) for the 931 tau-PET sessions with an accompanying CDR assessment within 2 years. Individual data points are plotted and randomly jittered to aid visualization. Group means accounting for age are plotted as horizontal dashed lines illustrating increases in TSS with worsening CDR score as expected.

The spatial extent of tau pathology was quantified using the tau spatial spread (TSS) metric (see Online Methods). Modeling by CDR status, there are significant group differences in TSS between CDR=0 (M=0.049, SD=0.076) and CDR > 0 (M = 0.313, SD=0.294), t(929) = 21.59, *p* < .0001, 95% CI [0.24,0.29]. Figure 13 illustrates this increase in TSS with increasing CDR across the 931 tau-PET sessions for which a CDR assessment was obtained within 2 years, highlighting the differences along the AD continuum.

Age-related differences in TSS were modeled across CDR stages using the same mixed-effects framework. Results revealed a significant main effect of age (β = 0.00265, SE = 0.00044, t(880) = 6.10, p < .0001), indicating that TSS increased with age. Significant effects of all CDR categories were observed: CDR 0.5 (β = 0.180, SE = 0.012, t(918) = 14.42, p < .0001), CDR 1 (β = 0.400, SE = 0.017, t(904) = 23.90, p < .0001), and CDR 2 (β = 0.629, SE = 0.036, t(465) = 17.36, p < .0001), suggesting that participants with cognitive impairment had greater TSS than unimpaired participants. The omnibus age × CDR interaction was significant, (F(3, 684) = 69.95, p < .0001). Follow-up contrasts revealed significant interactions for CDR 0.5 (β = −0.011, SE = 0.001, t(781) = −7.97, p < .0001), CDR 1 (β = −0.024, SE = 0.002, t(858) = −12.92, p < .0001), and CDR 2 (β = −0.015, SE = 0.003, t(436) = −4.35, p < .0001), indicating that the effect of age on TSS differed across impairment levels. Estimated marginal means confirmed significant differences between all CDR groups (CDR 0 = 0.052 ± 0.004, CDR 0.5 = 0.232 ± 0.012, CDR 1 = 0.452 ± 0.016, CDR 2 = 0.682 ± 0.036; Tukey-adjusted p < .0001).

#### White Matter Hyperintensities

Radiological findings, such as white matter hyperintensities (WMH), microbleeds, and infarcts, are commonly found in those with dementia and are characterized within the dataset. These measures and reads of interest do not undergo systematic QC and can be subject to high variability across longitudinal sessions. To evaluate associations with age and WMH across clinical stages, we fit a linear mixed-effects model with age, CDR stage (0, 0.5, 1+), and their interaction, accounting for the longitudinal design with a random effect for participant. WMH volume was log-transformed to normalize its distribution. The model revealed a significant main effect of age (β = 0.040, SE = 0.002, t(1333) = 21.52, *p* < .0001), indicating age-related increases in WMH. Compared to CDR 0, participants with CDR 0.5 had significantly greater WMH volume (β = 1.362, SE = 0.443, t(1458) = 3.07, *p* = .0022), while those with CDR 1+ showed a trend toward higher WMH volume (β = 1.324, SE = 0.756, t(1426) = 1.75, *p* = .0800). Additionally, there was a significant interaction between age and CDR 0.5 (β = −0.016, SE = 0.006, t(1456) = −2.71, *p* = .0068), suggesting that the age-related increase in WMH was attenuated in this group. The interaction between age and CDR 1+ was not statistically significant (β = −0.014, SE = 0.010, t(1438) = −1.42, *p* = .1571).

**Figure 14:**
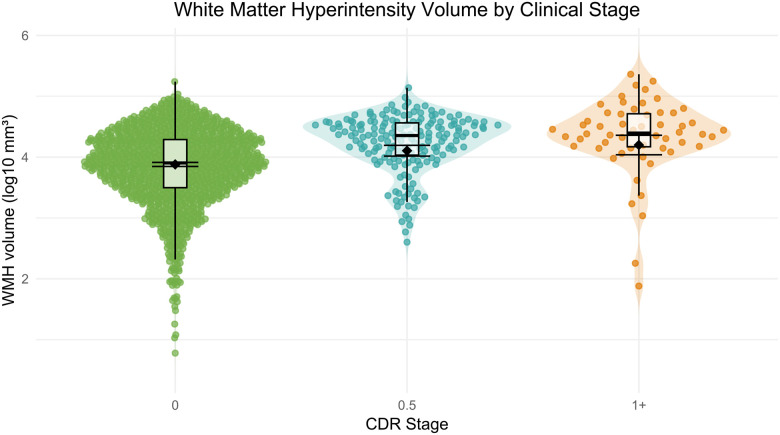
Violin and box plots show the distribution of log-transformed white matter hyperintensity (WMH) volume (log_10_ mm³) across Clinical Dementia Rating (CDR) stages: 0, 0.5, and 1+. Data drawn from the 1,491 MRI sessions with CDR assessments within 2 years. Embedded boxplots indicate the median and interquartile range of observed scores, with whiskers extending to 1.5× the interquartile range. Error bars show the 95% confidence intervals around the model-adjusted means. Colored dots represent individual data points while black diamonds indicate model-estimated marginal means from the linear mixed-effects model adjusting for age and accounting for repeated measures. Post hoc comparisons confirmed higher WMH volume in both CDR 0.5 and CDR 1+ vs. CDR 0 (*p* < .0001).

Post hoc comparisons of estimated marginal means (on the log-transformed scale) revealed that WMH volume differed significantly between CDR 0 and both CDR 0.5 (*p* < .0001) and CDR 1+ (*p* = .0004), but not between CDR 0.5 and CDR 1+ (*p* = .5672). The estimated marginal means (±SE) were: CDR 0 = 3.88 (0.02), CDR 0.5 = 4.11 (0.05), and CDR 1+ = 4.20 (0.08). These findings indicate that WMH volume increases with both age and clinical impairment, although the rate of age-related increase may vary by disease severity (figure 14).

#### Radiological Reads

##### Leukoaraiosis

Radiological ratings of WMH severity (also referred to as leukoaraiosis; categorized as “None,” “Mild,” and “Moderate” and “Severe”) and CDR status were evaluated using a chi-square test of independence. The “Moderate” and “Severe” leukoaraiosis categories were combined due to the low number of participants with severe WMH ratings. There was a significant relationship between WMH severity and CDR status, χ^2^(2, N = 794) = 19.80, *p* < .0001, indicating that the distribution of WMH scores differed across clinical groups. Post hoc comparisons with Bonferroni correction revealed that participants with CDR = 0 had a significantly higher-than-expected proportion of scans rated as “None” (30.7%, *p* = .0231), while participants with CDR > 0 had a significantly higher-than-expected proportion of “Moderate to Severe” ratings (18.6%, *p* = .0006). No significant deviations from expected frequencies were found for “Mild” WMH ratings in either group.

##### Cerebral Microbleeds

To examine the relationship between cerebral microbleeds (CMB) and clinical dementia status, we first binarized CMB ratings to reflect the presence (1) or absence (0) of microbleeds. A chi-square test revealed a significant association between CMB presence and Clinical Dementia Rating (CDR) category (χ^2^(1) = 10.76, *p* = 0.0010), with 17.1% of individuals with CMBs classified as impaired (CDR > 0) compared to 8.1% without CMBs. Fisher’s exact test confirmed this association (*p* = 0.0024; odds ratio = 2.34, 95% CI = [1.33, 4.04]). To account for age-related effects, we conducted a binary logistic regression predicting CMB presence from age and CDR category. CDR > 0 remained a significant predictor of CMB presence (log odds = 0.72 ± 0.27, *p* = 0.0083), corresponding to a 2.06-fold increase in the odds of having CMBs. Age showed a marginal association (log odds = 0.030 ± 0.015, *p* = 0.0539), indicating a trend toward increased CMB prevalence with advancing age. These results suggest that CMBs are more common among cognitively impaired individuals, independent of age.

##### Cerebral Infarcts

To examine the relationship between CDR status and the presence of cerebral infarcts, we conducted a Fisher’s exact test on a 2×2 contingency table. Cerebral infarcts were coded as “Yes” if any infarcts were detected (collapsing across both small and large infarct ratings) and “No” if none were identified. Results indicated no significant association between CDR status and infarct presence (Fisher’s exact test, *p* = 0.8192; odds ratio = 0.83, 95% CI [0.25, 2.23]). Although the observed frequencies were closely aligned with expected counts (Yates-corrected χ^2^ = 0.02, *p* = 0.8818), interpretation is limited by the small number of participants with both impaired CDR and infarcts (n = 5), which constrains statistical power and the precision of effect estimates.

### Biological Staging

Current research frameworks have devised classification or staging criteria for the characterization and diagnosis of AD according to amyloid, tau, and neurodegenerative biomarkers (A/T/N framework; (Jack Jr et al., 2018)). Recent updates have progressed towards the quantitative estimation of biological factors, incorporating amyloid positivity and tau-PET spread, leading to a 4-stage model of AD progression (Jack Jr et al., 2024) Here we apply this staging model to the KARI dataset to provide insight into the composition of participants along these biological criteria.

Analysis was run on participants who have a processed T1-weighted MRI, amyloid-PET, and a tau-PET scan, all acquired within 1 year of each other. PET biomarker cutoffs for staging were established using a Gaussian mixture model implemented via the *mclust* package (Fraley et al., 2012) in R (R Core Team, 2023) within the RStudio environment (Posit Team, 2023), consistent with prior applications of GMM for deriving amyloid and tau-PET thresholds in AD research (Quattrini et al., 2023; Therriault et al., 2021). This included 656 unique participants with a total of 812 matched scan sessions. Of these, 547 were amyloid negative according to a Gaussian mixture model (GMM) of whole-brain Centiloid values which identified 2 components with a Centiloid cut point of 18.42. To determine staging, we quantified tau standardized uptake value ratio (SUVR) from a medial temporal lobe (MTL) meta-ROI and a neocortical (NEO) meta-ROI derived using FreeSurfer regions without partial volume correction (Villemagne et al., 2023). GMM in the MTL region identified two groups, positive and negative, with a cut point of 1.28. Similarly, a GMM in the NEO region identified three groups, negative, low, and high, with cut points at 1.23 and 1.42. Of the remaining 265 sessions, 144 sessions were classified as stage A: amyloid positive (mean = 50.41), MTL negative (mean = 1.13), NEO negative (mean = 1.10); 44 as stage B: amyloid positive (mean = 73.58) MTL positive (mean = 1.40), NEO negative (mean = 1.25); 29 as stage C: amyloid positive (mean = 88.23) MTL positive (mean = 1.51), NEO low (mean = 1.32); and 43 as stage D: amyloid positive (mean = 120.88), MTL positive (mean = 1.68), NEO high (mean = 1.98; Table 5). An additional 5 sessions were unclassified as amyloid positive, MTL negative, and NEO positive, possibly indicating an MTL-sparing subgroup.

### Healthy aging

The KARI dataset is enriched for the recruitment of preclinical AD participants encompassing the older adult lifespan. The dataset’s design specifically aims to track the progression of AD from the healthy adult stage through the initial phases of disease pathology. Consequently, a significant portion of the cohort consists of asymptomatic individuals who meet the criteria for a healthy control group in adult lifespan and aging research. Biological staging indicates that 434 participants have acquired 591 matched sessions of data, each with a T1-weighted MRI, amyloid-PET, and tau-PET scan collected within 1 year, that are both amyloid and tau-PET negative. Incorporating clinical data, 372 participants across 456 sessions are unimpaired with a CDR = 0 who remain unimpaired as of their most recent data acquired. When considering only MRI and amyloid-PET, 647 participants have 1248 matched sessions of data that satisfy the same criteria of having a T1-weighted and amyloid-PET acquired within 1 year and maintained a CDR of 0 across the study. This includes 301 participants with longitudinal data of at least 2 timepoints.

## Discussion

The Knight Alzheimer Research Imaging dataset is a significant, publicly accessible resource distinguished by its longitudinal depth, multimodal neuroimaging, and a strong representation of individuals in the preclinical stages of AD. Integrating over 6,000 imaging sessions with clinical, cognitive, and genetic assessments, KARI is uniquely suited to explore biomarker trajectories, neurodegeneration, and cognitive decline across the AD continuum. The integration of cognitively normal, preclinical, and symptomatic participants, tracked over time with a comprehensive imaging battery, makes this resource particularly valuable for research focused on the earliest phases of AD, including early detection and prevention strategies. This unique combination of features positions KARI to make significant contributions to understanding the sequence and interplay of pathological events driving cognitive declines and clinical onset.

Research from the Knight ADRC has substantially advanced understanding of AD biomarkers and their predictive value across disease stages, highlighting the importance of the preclinical phase and our ability to detect early vulnerabilities. Hassenstab et al. (2016) demonstrated that cognitively normal individuals with preclinical AD biomarkers performed worse on cognitive tests than biomarker-negative peers. Excluding these individuals from normative samples greatly reduced apparent age-related cognitive decline, suggesting a disease-driven component. Similarly, Sheline et al. (2010) found that cognitively normal *APOE E*4 carriers exhibit disrupted resting state functional connectivity before amyloid-β pathology detected by PET or CSF, indicating a genetically mediated network vulnerability. Longitudinal analyses by Schindler et al. (2021) revealed a nonlinear trajectory of Aβ accumulation, accelerating at a “tipping point” predictive of future cognitive decline. Plasma biomarkers, such as the Aβ42/Aβ40 ratio, offer scalable tools for identifying individuals nearing this threshold (Schindler et al., 2019). Complementary multimodal brain age models have shown promise as sensitive indicators of early pathological changes, with strong associations to tau, amyloid, and cognitive function in presymptomatic individuals (Millar et al., 2023).

KARI data have also clarified mechanisms linking amyloid and tau pathology, implicating neuroinflammation as a potential intermediary. Using diffusion MRI techniques, Wang et al. (2024) found elevated white matter neuroinflammation in cognitively normal individuals with both amyloid and tau pathology (A+T+), but not with amyloid alone (A+T-) suggesting inflammation facilitates tau propagation. Tau-PET imaging further supports this cascade. In cognitively normal individuals, tau-PET signal correlated with CSF amyloid but not CSF tau, positing amyloid as a prerequisite for tau aggregation (Gordon et al., 2016).

Tau pathology has emerged as a strong predictor of cognitive decline. Tau-PET outperforms amyloid-PET and structural MRI in explaining cognitive decline, particularly when combined with hippocampal atrophy and amyloid positivity (Aschenbrenner et al., 2018; Brier et al., 2016). Similarly, machine learning analyses integrating 27 biomarkers found certain phosphorylated tau measures (e.g., p-tau181, p-tau217) to be predictors of amyloid-PET status and future cognitive decline (Meeker et al., 2024). Within amyloid-positive individuals, tau-PET and CSF tau levels are the most informative indicators of cognitive impairment. Additionally, the tauopathy metric, is highly effective at identifying early tau accumulation and is associated with subtle cognitive decline in preclinical stages (Mishra et al., 2017). Doering et al. (2024) further distinguishing tau burden and tau spatial spread, showing that tau spreads even in early disease stages, while later stages involve concurrent progressive tau spread and local accumulation, an insight with implications for stage-specific therapeutic targeting.

KARI data have also informed evaluation of novel AD therapies, anchoring treatment effects to meaningful clinical outcomes. For instance, recent analyses assessed the clinical relevance of slowing CDR Sum of Boxes progression in response to disease-modifying treatments, linking biomarker change to clinical benefit (Hartz et al., 2025).

Together, these findings illustrate that AD progression reflects a dynamic interplay among imaging, genetic, and fluid biomarkers. Tau pathology remains most closely tied to cognitive outcomes, whereas amyloid burden and APOE ε4 status inform disease onset and risk. Collectively, these results underscore the value of multimodal biomarkers for early detection, risk stratification, and precision-targeted intervention, highlighting the broad impact of the KARI dataset in advancing AD research.

The Charles F. and Joanne Knight Alzheimer Disease Research Center is an essential resource advancing the understanding and treatment of AD. Its longstanding commitment to longitudinal, multimodal data collection and open sharing has enabled research spanning diagnostic development, therapeutic evaluation, and elucidation of disease mechanisms. Continued support for such longitudinal studies is critical to address the complexity of neurodegenerative disorders. The KARI dataset provides a powerful foundation for hypothesis generation, biomarker validation, and refinement of disease models, supporting investigation across the healthy lifespan, preclinical stages, and symptomatic progression. Collectively, these efforts accelerate discovery and advance precision approaches in Alzheimer research.

## Online Methods

### Broad methodological considerations

The Knight ADRC has established this KARI dataset as a comprehensive and longitudinal scientific resource aimed at advancing the understanding of AD through multimodal neuroimaging and associated clinical, genetic, and biomarker data. This paper provides a detailed characterization of the KARI dataset, with a particular focus on the acquisition and processing of the 6,217 neuroimaging sessions collected across 1,645 participants as of data freeze 25 (released November 30^th^, 2024). The subsequent sections of this Method section describe the data acquisition procedures for magnetic resonance imaging (MRI) and positron emission tomography (PET), detail the quality control protocols implemented, outline the processing pipelines for structural MRI (including FreeSurfer analysis and derived measures), and PET imaging (including amyloid and tau quantification), and finally, describe the methods used for data storage and access.

### Participants and Recruitment

Participants were recruited through ongoing studies affiliated with the Knight ADRC at Washington University in St. Louis, with a targeted focus on middle aged individuals to capture the onset of preclinical or presymptomatic stages of AD. Recruitment is ongoing for both new and enrolled participants, targeting those at increased genetic risk for AD (e.g., having a parent with symptomatic AD before age 80) and those without a known family history. Recruitment methods include word of mouth, public service announcements within the greater St. Louis area, and physician referrals (~17%). Participants are referred to the Imaging Core via the Clinical Core, with referrals managed and tracked through REDCap, a secure electronic database. Imaging is typically scheduled within three months of clinical assessment.

All participants, or their legally authorized representatives, provided written informed consent prior to enrollment, in accordance with the Declaration of Helsinki. Study procedures were approved by the Washington University Institutional Review Board (IRB), Human Research Protection Office (HRPO), and the Radioactive Drug Research Committee (RDRC). Imaging protocols fall under an IRB-approved observational study registered on ClinicalTrials.gov (NCT04579120), with HRPO approval number 201906009. Inclusion and exclusion criteria are reviewed prior to each imaging session. If a participant is deemed ineligible for a specific modality at a given visit, they may continue with other procedures for which they qualify, with eligibility reevaluated at future visits. Regulatory approvals are actively maintained and updated.

### Clinical Measures

As part of their participation, all Knight ADRC participants undergo a comprehensive clinical and cognitive evaluation. This battery incorporates the Uniform Data Set (UDS (Morris et al., 2006)) that includes widely used instruments such as the Geriatric Depression Scale (GDS;(Sheikh & Yesavage, 2014)) and the CDR, as well as additional tests such as the Mini-Mental State Examination (MMSE;(Folstein, 1975)), among others. The CDR serves as the primary measure of symptomatic status in our studies. The clinical assessments include a structured interview between participants and an informed family member or caregiver to evaluate their memory, orientation, judgement and problem solving, community affairs, hobbies, and personal care. Scores are determined according to a five-point scale relating to disease severity such that 0 = normal/no impairment, 0.5 = very mild, 1 = mild, 2 = moderate, and 3 = severe dementia.

### Cognitive Measures

The Knight ADRC remains consist with other national ADRCs by conducting cognitive testing in accordance with the (UDS) (Beekly et al., 2007). This testing generates comprehensive cognitive data by using a range of common neuropsychological tests. In addition to the UDS measures, we also administer additional tests such as the Free and Cued Selective Reminding (FCSR) test (Grober et al., 2000). From these data, the Knight Preclinical AD Cognitive Composite (Knight PACC) is derived, along with four domain-specific composites: episodic memory, semantic memory, attention and processing speed, and working memory (McKay et al., 2024). The Knight PACC is a summary measure designed to sensitively detect early cognitive changes resulting from preclinical AD pathology. Briefly, it is a modified version of previously described PACCs (Donohue et al., 2014) that excludes MMSE from its calculation due to the low variability in MMSE scores and known ceiling and floor effects (McKay et al., 2024). While the Knight PACC can be requested as a computed variable, it can also be derived. The Knight PACC is computed as the arithmetic mean of the z-scores associated with the free recall score of the free and cued selective reminding test (Grober et al., 1998), the total correct score from the Digit Symbol subset of the WAIS-R (Wechsler, 1981), the total completion time from the Trail Making Test Part B (Armitage, 1946), and the total correct score from the Animal Naming Test (Goodglass & Kaplan, 1983). Importantly, z-scores should be calculated relative to cognitively unimpaired individuals within the sample of interest, or relative to baseline cognitive visit in subsamples without unimpaired individuals. Before calculating the PACC score, standardized scores for the Trail Making Test are reverse scored to ensure that higher scores consistently represent better task performance. See McKay et al., 2024 for detailed discussion of these variables.

Additional, non-imaging focused analyses were run to examine the relationship between clinical status and cognition. Using a linear mixed-effects model we predicted PACC scores from CDR group (0, 0.5, 1, or 2+), while adjusting for age, education, sex, and APOE ε4 status. This analysis encompassed 11,622 cognitive assessments from 2,364 unique individuals with CDR score matched to the closest clinical assessment within two years. Cognitive performance declined significantly with increasing CDR severity. Estimated marginal means (± SE) for PACC scores were −0.03 ± 0.013 for CDR 0, −0.43 ± 0.015 for CDR 0.5, −0.98 ± 0.020 for CDR 1, and −1.29 ± 0.067 for CDR 2+. All pairwise group comparisons were statistically significant after Tukey correction (ps < .0001). These results indicate a robust and graded association between worsening clinical status and cognitive impairment, even after adjusting for relevant demographic and genetic factors, and accounting for within-subject variability over time using Kenward–Roger degrees of freedom (figure S1).

### Neuroimaging Procedures

The KARI imaging variables consist of software-processed data, with radiological reads for a subset of structural MRI data. The software-processed neuroimaging data is overseen and quality-controlled by the trained experts of the Imaging Core Laboratories at Washington University School of Medicine Neuroimaging Laboratories Research Center. All T1-weighted MRI data is processed through the FreeSurfer software suite for cortical reconstruction and volumetric segmentation, while PET images were processed using both a FreeSurfer dependent PET Unified Pipeline (PUP) and an MRI-free processing pipeline. Workflows for both processes are described in the Online Methods section. T2-FLAIR scans are also processed through an automated pipeline using SPM’s lesion growth algorithm (LGA) within the Lesion Segmentation Tool (LST) for white matter hyperintensity (WMH) segmentation (Schmidt et al., 2012). To date, 1,929 sessions across 1,083 unique individuals have associated WMH total volume data. Additionally, T1-weighted, T2-FLAIR, and T2-star/SWI MRI are reviewed by a board-certified neuroradiologist to identify and rate any radiological finding of interest (e.g., assess atrophic process, leukoaraiosis or white matter hyperintensities, microbleeds, infarcts, etc.). Here we present summary measures of interest to ADRD research derived from structural and PET imaging and separated by CDR status.

#### Initial Quality Control at Acquisition

To ensure the quality of imaging data, the KARI Imaging Core implements a multi-layered quality control process on de-identified data before processing or analysis. This begins with a quality assurance and preventative maintenance program for all research scanners, conducted through collaborative efforts between Siemens engineers and the chief technologists for MRI and PET. Longitudinal scanner stability is monitored via quarterly phantom scans. At the time of acquisition, study coordinators confirm imaging sequence parameters, and acquired images are immediately assessed for motion artifacts, with rescans performed and documented as needed both on study forms and electronically during archiving. Subsequent quality control measures are integrated into each stage of the downstream processing pipeline, described in further detail below.

### MRI Acquisition:

All neuroimaging data were acquired at dedicated research imaging facilities within Washington University School of Medicine. Standardized procedures were implemented to ensure participant comfort, including clear instructions and head positioning with appropriate padding and restraints to minimize motion during scans. The environment was controlled to maintain consistency across projects and acquisition sessions.

MRI data included in this characterization were predominantly acquired on Siemens 3 Tesla (3T) platforms, including the TIM Trio, Biograph mMR (PET-MR system), Prisma fit, and MAGNETOM Vida. A smaller portion of historical data, acquired on Siemens 1.5T scanners (Sonata, Vision), is available as part of the broader KARI data release but is not specifically characterized in this report due to differences in field strength and post-processing methods. The use of multiple scanner platforms over the extended duration of this longitudinal study necessitates careful consideration of inter-scanner variability, which has been addressed through harmonization efforts and quality control procedures detailed below.

A comprehensive suite of MRI sequences was acquired, including T1-weighted (T1w) anatomical scans, T2-weighted (T2w), T2-weighted Fluid-Attenuated Inversion Recovery (T2-FLAIR), T2-star-weighted (T2*) or Susceptibility-Weighted Imaging (SWI), diffusion-weighted imaging (dMRI), Arterial Spin Labeling (ASL), and resting-state functional MRI (rs-fMRI) utilizing Blood Oxygenation Level Dependent (BOLD) contrast. The T2*/SWI sequences, while varying slightly in specific protocol parameters over time due to optimization and harmonization efforts, were primarily implemented for the detection of cerebral microhemorrhages and related susceptibility effects; data from these related sequences are aggregated under the T2*/SWI category for reporting purposes.

#### MRI Scanner Harmonization

Given that MRI data within the KARI dataset were acquired across several Siemens platforms (including the TIM Trio, Biograph mMR, Prisma fit, MAGNETOM Vida, Sonata, and Vision, operating at both 1.5T and 3T field strengths), post-acquisition harmonization measures should be implemented to ensure consistency within an analysis. We tested hippocampal volume concordance in a subset of participants to assess the comparability of volumetric measures obtained from our primary 3T scanners, the Siemens TIM Trio and the Siemens Biograph 3T mMR (PET-MR). Sixty-nine participants (mean age 65.9 years, predominantly cognitively normal individuals) underwent MRI on both the Trio and mMR within a two-week interval. Volumetric segmentation of brain regions of interest, including the left and right hippocampus, was performed using FreeSurfer v5.1. Concordance correlation coefficients (CCCs) were calculated to assess the agreement between the two scanners for raw hippocampal volumes. Rank-based CCCs demonstrated excellent reproducibility for both left (CCC = 0.92, 95% CI [0.86, 0.95]) and right (CCC = 0.91, 95% CI [0.86, 0.95]) hippocampal volumes. These values are consistent with reported test-retest reliability within the same scanner. Based on these findings, and in line with established neuroimaging harmonization practices, we proceeded with the combined analysis of data acquired from these 3T platforms, while acknowledging the potential for residual site-related effects as a factor to consider in downstream analyses. Similar caution should be taken when analyzing data collected across scanners. Data acquired on the 1.5T scanners (Sonata and Vision) are not characterized within this report due to the age of the scans and the lower field strength but are available as part of the KARI data release.

### PET Acquisition

PET data were acquired on several Siemens scanners, including dedicated PET scanners Siemens HR+, Biograph mCT, and Biograph Vision, as well as a Biograph mMR hybrid PET/MR scanner. Standardized procedures were followed for all PET acquisitions. An intravenous catheter was placed for tracer administration. Following tracer injection (a single IV bolus), participants rested quietly during the prescribed uptake period in a controlled environment. Scans were acquired with appropriate head positioning and motion restraint. Dynamic acquisitions involved list-mode data collection over specified durations, allowing for flexible frame reconstruction. Scans do not undergo post-reconstruction smoothing but use standard decay, scatter, and randoms corrections based on scanner model.

#### Amyloid-PET Tracers

Three amyloid-PET tracers were used: ¹¹C-Pittsburgh Compound B (PiB) (Klunk et al., 2004), ^18^F-Florbetapir (FBP) (Clark et al., 2011), and ^18^F-Florbetaben (FBB) (Rowe et al., 2008). PiB was administered at a dose of approximately 13.94 mCi (SD=3.76), with scans acquired 30–60 minutes post-injection. FBP was administered at a dose of approximately 9.92 mCi (SD=0.63), with scans acquired 50–70 minutes. FBB was administered at approximately 8.14 mCi (SD=0.36), with scans acquired 90–100 minutes post-injection. For scans acquired using the Biograph mMR prior to the VE11P software update, CT-based attenuation correction was performed (Su, Rubin, et al., 2016). Amyloid SUVRs are calculated using a cerebellum reference region, further scaled to a global Centiloid composite calculated from the mean lateral orbitofrontal, medial orbitofrontal, precuneus, rostral middle frontal, superior frontal, superior temporal, and middle temporal regions (Klunk et al., 2015). The choice of tracers and acquisition windows reflects established protocols for amyloid quantification and facilitates comparison across a broad range of AD research studies.

#### Tau-PET Tracer

^18^F-Flortaucipir (FTP) was used for in-vivo imaging of tau pathology (Chien et al., 2013). FTP was administered at a dose of approximately 8.98 mCi (SD=0.82), with dynamic scans acquired 80–100 minutes post-injection. Quantitative analyses were performed using the cerebellum-cortex as the reference region for SUVR calculation.

#### FDG-PET Tracer

FDG-PET sessions were conducted in a subset of participants using the standard [18F]-Fluorodeoxyglucose (FDG) tracer (Phelps et al., 1979). In total, 173 separate FDG-PET sessions were conducted across 149 unique individuals, with 24 of these individuals having two timepoints of longitudinal data separated by an average lag of 2.04 years (SD = 1.73). While these data contributed valuable insights toward glucose metabolism in AD (Goyal et al., 2023; Goyal et al., 2020), given the relatively small proportion of FDG-PET acquired, it is not considered a primary variable of interest in this characterization.

### MRI Processing

T1-weighted structural head MRI scans are segmented into cortical and subcortical regions of interest (ROIs) using the “recon-all” procedure from FreeSurfer version 5.3 and 7 (Massachusetts General Hospital, Charlestown, MA)(Fischl, 2012). In general, this processing pipeline involves motion artifact correction, intensity normalization, a hybrid watershed and surface deformation procedure for brain extraction (Ségonne et al., 2004), and affine registration to the MNI-305 atlas for intensity-based identification of the white matter surface and overlaying cortical gray surface (Fischl et al., 1999). Cortical and subcortical ROIs are labeled based on the gyral and sulcal structures using the Desikan-Killany atlas (Desikan et al., 2006). Subject-specific regional thickness, volumetric, and surface area measures from this workflow are available in the KARI data set.

Following processing, FreeSurfer segmentation and surface image output is manually inspected by trained Imaging Core research staff within the Neuroimaging Laboratories Research Center. During this process, the rater will identify all visual errors in the cortical parcellation and subcortical segmentation that meet certain criteria. Errors meeting criteria undergo intervention via manual edits and reprocessing of the corrected data to update FreeSurfer’s quantitative measures. Error identification and manual intervention procedures follow FreeSurfer recommendations for quality control of output but will be described here.

FreeSurfer errors generally fall into two categories: inclusions and exclusions. Inclusions involve non-brain regions, such as the dura or skull, being included in either the surface or ROI labeling. Exclusions are brain regions excluded from either surface or ROI labeling. Generally, up to three attempts at correcting errors will be performed if the errors persist even after manual intervention. Following the third attempt, the image will either pass with edits to the output or fail quality control. Common errors requiring manual intervention include lateral ventricles segmented as white matter if larger than 300 mm^3^, subcortical white matter exclusions in the temporal regions if larger than 60 mm^3^, dural inclusions on the cortical grey surface or gray matter exclusions if larger than 120 mm^3^, and subcortical segmentation exclusions if greater than 120 mm^3^.

Volumetric output from two different versions of FreeSurfer is included in the dataset. Prior work has shown variability in output from the same version run on different computing platforms or with different MRI acquisition parameters, and even across scan-rescan datasets (Gronenschild et al., 2012; Han et al., 2006; Jovicich et al., 2009; Morey et al., 2010). The KARI dataset combines results from version 5.3 with the HCP patch applied, run on a CentOS 7-based platform, and containerized releases of FreeSurfer version 7. The FreeSurfer 7 dataset includes output from versions 7.3.2 and 7.4.1 which are described by the developers as providing identical results to other FreeSurfer 7 versions. Combining different FreeSurfer software versions for volumetric processing has been evaluated with mixed results. While some work has suggested that combining values from different versions of the software might affect outcomes in inter-group comparisons (Filip et al., 2022), others have shown that versions can be combined reliably in AD cohorts (Chepkoech et al., 2016). As the reprocessing of the KARI dataset in FreeSurfer 7 is ongoing, there is a subset of newer scans that have not received FreeSurfer 5.3 processing and a subset of older scans that have not yet received FreeSurfer 7 processing. We provide FreeSurfer output from both versions where it is available, and we suggest taking the FreeSurfer version number into account when performing analyses.

For volumetric analyses of cortical regions derived from FreeSurfer, we note that these measures can be influenced by individual head size. Following the recommendations of Buckner et al. (2004), intracranial volume (ICV) can be used for normalization to facilitate accurate comparisons. This correction is not typically applied to cortical thickness measures, which are largely independent of head size. As the appropriate ICV correction is sample-specific and dependent on the included participants, the KARI data release provides raw FreeSurfer volume outputs, allowing investigators to perform ICV correction tailored to their specific analytical cohorts.

#### White Matter Hyperintensities

WMH volumes were extracted from both T1 and FLAIR MRI scans using version 1.2.3 of the Lesion Segmentation Toolbox (LST) within SPM8 (Schmidt et al., 2012). These volumes serve as a measure of white matter damage. Detailed information can be found in the LST user manual provided with the software download (https://www.applied-statistics.de/LST_1.2.3.zip). The binary lesion maps were aligned with MNI space, and the outputs maintained the same resolution as the T1 scans, typically 1×1×1 mm. Additional correction for intracranial volume was not applied. Note that the automated LST method may overestimate WMH lesions compared to manual tracing. Data releases include total WM hyperintensity volume in cubic millimeters (mm^3^).

#### Radiological Reads

T2/FLAIR scans were qualitatively assessed for white matter damage by a neurologist, focusing on periventricular and deep white matter. Radiologists at Washington University interpret all local KARI MRI scans for the identification of aging patterns and pathologies such as small vessel disease, white matter hyperintensities, microhemorrhages, and previous strokes. This includes the assessment of amyloid-related imaging abnormality (ARIA) using the FDA guidelines (vasogenic edema, microhemorrhages (<10 mm), macrohemorrhages (>10 mm), subarachnoid hemorrhage, and hemosiderosis). MRI sessions are uploaded to the central CNDA archive where readers can review the images and enter their readings via customized reporting tools. Abnormal findings are flagged and referred to the Imaging Core Leader, Dr. Benzinger, and study Principal Investigator, Dr. Morris (for Knight ADRC studies), for further action. A HIPAA-compliant workflow developed in collaboration with Barnes-Jewish Hospital permits scans with abnormal findings to be incorporated into the participant’s electronic medical record, if desired. Neuroradiological interpretation data logged in the CNDA is transmitted to the Biostatistics Core with each KARI data release.

Neuroradiologists determine cortical atrophy and aging changes through standard clinical assessments. Below, we describe the main reads presented as ordinal or categorical variables in our comprehensive report, and more detailed diagnosis information and additional ratings (e.g., cortical and hippocampal atrophy) can be found in the data freeze and data dictionary.

Leukoaraiosis (white matter hyperintensities) assessment used the Fazekas score, ranging from 0 to 3 (0 = None, 1 = Mild, 2 = Moderate, 3 = Severe). Details on the approach can be found in (Fazekas et al., 2002). Similarly, analyses used groups combining the Moderate and Severe groups (i.e., “None”, “Mild”, and “Moderate to Severe”).

Cerebral infarcts, categorized either as small (<1cm) or large (≥1cm), were scored using the method described by (Price et al., 1997) ranging from 0 to 3 (0 = None, 1 = Mild, 2 = Moderate, 3 = Severe). Because of the scarcity of this type of finding, all infarcts regardless of size were grouped as present versus absent in analyses (see Additional Statistical Analyses section below).

A range of cerebral microbleeds, defined as lesions <10mm on T2*-GRE or SWI, are reported as 0, 1, 5, or 11. We presented these categories as None for 0, Mild for 1, and Moderate to Severe combining scores 5 and 11.

### PET Data Processing and Quantification

PET data processing aims to provide accurate and reliable quantification of tracer uptake in various brain regions. The KARI pipeline includes both MR-dependent and MR-free processing streams, as well as corrections for physical factors that can affect PET signal.

#### MR-dependent PET Processing

ROI-based PET quantification is performed using the PET Unified Pipeline (PUP, https://github.com/ysu001/PUP) with FreeSurfer ROIs (Su et al., 2015; Su et al., 2013). The PET data is first smoothed to a common full-width half-max (FWHM) of 8mm to minimize inter-scanner differences (Joshi et al., 2009). For dynamic PET acquisitions, frames are motion corrected using affine registration methods (Eisenstein et al., 2012; Hajnal et al., 1995). Motion-corrected PET data is then registered to the structural T1 head MRI using a cross-modal vector-gradient algorithm (Rowland et al., 2005). Time activity curves for each FreeSurfer ROI are extracted from the co-registered, motion-corrected PET data and summed over the tracer-specific post-injection quantification time window: 30–60 mins for PiB, 50–70 mins for FBP, 90–110 mins for FBB, 40–60 mins for FDG, and 80–100 mins for FTP. Standardized uptake value ratios (SUVRs) are calculated for each FreeSurfer ROI using a tracer-specific reference region. To date, all tracers in the KARI data set use cerebellar grey matter as the reference region. For dynamic PET data, non-displaceable binding potentials (BP_ND_) are calculated for the same window using a Logan graphical analysis (Logan et al., 1996; Mintun et al., 2006).

Given the low spatial resolution of PET imaging and longitudinal changes in atrophy for both normal aging and AD, partial volume effects (PVE) can potentially bias SUVR and BP_ND_ measures. In cases where the T1 structural head MRI was acquired within one year of the PET data, PUP-derived SUVRs and BP_ND_ are corrected for PVE using a regional spread function (RSF) (Rousset et al., 1998) based approach (Su et al., 2015). For cases where the MRI was acquired within 1–2 years from the PET, a neuroradiologist will review both the PET and MRI scans to determine whether atrophy in the MRI could significantly impact PET quantification. All cases greater than 2 years do not undergo RSF correction.

To measure global amyloid burden, the volume-weighted SUVRs or BP_ND_ for the bilateral precuneus, superior frontal, rostral middle frontal, lateral and medial orbitofrontal, and superior and middle temporal regions are averaged to define a mean cortical SUVR (MCSUVR) or BP_ND_ (MCBP).

For measuring tau burden, several measures are available. A global cortical summary measure of tau accumulation, tauopathy, is defined as the average of RSF-corrected SUVRs for bilateral entorhinal cortex, inferior temporal, lateral occipital, and the amygdala (Mishra et al., 2017). A measure of the spatial extent of tau pathology, tau spatial spread (TSS), is derived by calculating the proportion of voxels with abnormal tau pathology from tau-PET voxel-wise SUVRs. Abnormal tau pathology is identified by first z-scoring the voxel-wise tau-PET SUVR image relative to a group of healthy, young controls. The resulting z-scores are masked to include cortical regions, the hippocampus, and the amygdala. Voxels that surpass a significance threshold of z=1.96 are then used to compute the TSS measure (Doering et al., 2024). A final measure of tau pathology is provided that aligns with the regions used in the CenTauR measure. Volume-weighted SUVRs for the medial temporal (MTL) and neocortical (NEO) composite regions are calculated and provided for combining KARI data with other data sets using the same CenTauR composite regions (Villemagne et al., 2023).

#### MR-free PET Processing

Structural head MRI scans are typically acquired contemporaneously with PET to assist with creating ROIs for quantitative analyses. Such analyses though become challenging if the structural MRI is not obtained, such as when a participant is claustrophobic, or if the acquired MRI is deemed unsuitable for creating ROIs, such as an MRI with severe motion artifacts. For all amyloid-PET data, KARI uses an implementation of the MR-free PET processing pipeline (Landau et al., 2023) to calculate a global measure of amyloid burden. PET data are first corrected for inter-frame motion and summed over the window of interest as in our MR-dependent PET processing. The summed PET image is then spatially normalized to a tracer-specific PET template image using SPM12’s normalization module. The normalized image is then smoothed to a FWHM of 8mm using a Gaussian kernel, and a non-PVE corrected cortical SUVR is calculated from the standard Centiloid cortical mask with the whole cerebellum as the reference region.

#### Partial Volume Correction

Due to the inherent lower spatial resolution of PET imaging, measured signal intensities can be affected by partial volume effects (PVE), which are influenced by region size, shape, and image resolution. In longitudinal studies of aging and neurodegenerative diseases like AD, brain atrophy further complicates PVE. To mitigate these distortions in FreeSurfer-processed PET data, we employ a regional spread function (RSF)-based partial volume correction technique (Rousset et al., 1998; Su et al., 2015). Our prior work has demonstrated the efficacy of this RSF method in improving PET quantification and enhancing the detection of longitudinal changes in amyloid burden (Su, Blazey, et al., 2016; Su et al., 2015). Consequently, standard FreeSurfer-based PET processing in the KARI pipeline includes outputs both with and without RSF correction. However, manually defined region-of-interest (ROI) based PET processing does not incorporate PVE correction due to the lack of the necessary detailed anatomical information for the RSF approach. Similarly, voxel-wise SUVR images are also provided without partial volume correction. For analyses utilizing FreeSurfer-processed PET data, we recommend using the RSF-corrected values. However, to avoid data loss and potential biases causing FreeSurfer processing failures, if combining FreeSurfer-based and manual ROI-based PET data, we advise using the non-partial volume corrected FreeSurfer outputs. Combining RSF-corrected FreeSurfer data with non-corrected manual ROI data is strongly discouraged.

### Derived Composite Measures

Beyond basic regional quantification, the KARI dataset includes several higher-level derived neuroimaging outcome measures that are commonly used in AD research to summarize complex patterns of pathology or neurodegeneration.

#### T1-Based Structural Composites

To facilitate comprehensive analyses, the KARI dataset includes several derived composite measures calculated from regional FreeSurfer outputs (https://surfer.nmr.mgh.harvard.edu/fswiki/MorphometryStats). These include global measures such as Whole Brain Volume (sum of cortical gray matter, cortical white matter, and subcortical gray matter), Total Cortex volume (sum of left and right hemispheres), Total Cortical White Matter volume (sum of left and right hemispheres), and Subcortical Gray matter volume (aggregate volume of thalamus, caudate, hippocampus, amygdala, nucleus accumbens, ventral diencephalon, and substantia nigra). Additionally, Total Ventricular Volume is calculated as the sum of the volumes of the left and right lateral, inferior lateral, third, fourth, and fifth ventricles.

Of relevance to characterizing neurodegenerative changes in AD, the dataset also features a pre-computed Cortical Signature Thickness measure (‘CortSig_Thickness’). This measure, as described in Dincer et al. (2020), represents a weighted average of cortical thickness values within brain regions identified as most vulnerable to early neurodegeneration in late-onset AD. As presented in the Results section, this Cortical Signature Thickness measure demonstrates significant associations with CDR and age, highlighting its utility as a marker of disease progression within the KARI cohort.

#### Amyloid-PET

To facilitate the comparison of amyloid-PET burden across different tracers and studies, we utilize the Centiloid scale, a standardized, continuous measure developed by the Centiloid Working Group (Klunk et al., 2015). Given the inherent variability in amyloid-PET imaging due to tracer-specific properties, acquisition parameters, and analysis workflows, the Centiloid scale provides a common metric for global amyloid deposition derived from mean cortical standardized uptake value ratios (SUVRs). In the KARI dataset, Centiloid values are generated using an MR-free PET processing pipeline. To convert our MR-free cortical SUVR to Centiloid, we performed a Level-2 Centiloid calibration using the publicly available GAAIN calibration datasets for FBB and PiB and the baseline DIAN-TU-001 dataset for FBP. The Level-2 calibration produced the following conversion equations:

Suppl. Eq. 1 $${CL}_{PiB,30-60min}=103.87\times \text{CorticalSUVR}-105.23$$

Suppl. Eq. 2 $${CL}_{\text{Florbetaben},90-110min}=202.97\times \text{CorticalSUVR}-194.32$$

Suppl. Eq. 3 $${CL}_{\text{Florbetaphir},50-70min}=176.17\times \text{CorticalSUVR}-189.99$$

#### Tau-PET

Several quantitative metrics are derived from tau-PET scans to characterize the burden and distribution of tau pathology. One method of global tau burden quantification is included in the KARI dataset via the summary measure termed ‘tauopathy’ (sometimes referred to as tau index or TI), as defined in (Mishra et al., 2017). This metric represents the arithmetic mean of partial volume corrected SUVRs derived from ^18^F-Flortaucipir PET within four key bilateral regions of interest identified using FreeSurfer: amygdala, entorhinal cortex, inferior temporal gyrus, and lateral occipital cortex. This composite measure offers a robust index of overall tau accumulation across regions known to be affected in the early stages of AD. Tauopathy is calculated during post-processing for all participants with adequate tau-PET output and is provided in each data release.

To quantify the spatial extent of tau pathology as measured by ^18^F-Flortaucipir PET, we utilize a metric termed tau spatial spread (TSS;(Doering et al., 2024)). This method involves analyzing voxel-wise tau-PET SUVR images. Each SUVR image is normalized relative to a group of healthy, young controls by converting voxel values to z-scores. These z-score maps are then masked to include key regions vulnerable to early tau accumulation, specifically cortical areas, the hippocampus, and the amygdala. TSS is calculated as the proportion of voxels within this masked region that exceed a predefined significance threshold (z > 1.96). This metric provides a quantitative measure of the extent of abnormally elevated tau across these anatomically relevant regions, offering insights into the staging and progression of tau pathology in vivo, complementary to regional or global tau summary measures.

#### Biological Staging Meta-ROIs

For classifying participants using a biological staging criteria, we derived regional tau-PET standardized uptake value ratios (SUVRs) from ^18^F-Flortaucipir PET images, focusing on regions representative of early and later-stage tau pathology (Jack Jr et al., 2024). Specifically, we compiled a sub-sample of our data consisting of all participant-matched sessions that included a 3T-MRI, amyloid-PET, and tau-PET acquired within 1 year. Two tau composites were generated using volume-weighted mean SUVRs from FreeSurfer-defined ROIs resulting in a Medial Temporal Lobe (MTL) tau composite and a Neocortical (NEO) tau composite.

The MTL tau composite was generated by averaging the non-partial volume corrected SUVR values within the bilateral entorhinal cortex, parahippocampus, and amygdala. These regions are known to exhibit early tau accumulation in the AD pathological cascade. The specific FreeSurfer labels corresponding to these regions were used to extract the relevant PET quantification data for each participant and a cutoff of 1.28 was determined using a Gaussian mixture model to identify two groups corresponding to tau negative and tau positive.

The NEO tau composite was generated by averaging the non-partial volume corrected SUVR values across a set of neocortical regions demonstrating tau pathology in later stages of the disease. These regions included the cuneus, precuneus, inferior temporal, middle temporal, superior temporal, banks of the superior temporal sulcus, inferior parietal, superior parietal, isthmus cingulate, lateral occipital, lingual, posterior cingulate, and supramarginal. Again, the corresponding FreeSurfer labels were utilized for regional data extraction for each participant and cutoffs of 1.23 and 1.42 were determined using a Gaussian mixture model to identify three groups corresponding to tau negative, tau moderate, and tau high respectively.

Additionally, we calculated amyloid positivity using a Centiloid threshold of 18.42 as determined using a Gaussian mixture model of all participants within the sub-sample to identify positive and negative groups. Using these amyloid and regional tau measures, we then classified participants into biological stages. This framework categorizes individuals into four main groups: A = amyloid positive, MTL tau negative, NEO tau negative; B = amyloid positive, MTL positive, NEO negative; C = amyloid positive, MTL positive, NEO moderate; and D = amyloid positive, MTL positive, NEO high. This approach allowed us to explore the distribution of our participants across the biological continuum of Alzheimer disease, providing insights into the relationship between amyloid and regional tau deposition in our KARI cohort. Tau MTL and NEO estimates were created using KARI data freeze 25 for this report and will be made publicly available in future data releases.

### Additional Statistical Analyses

Statistical methods employed within the results section of this paper to summarize the KARI dataset and illustrate defining characteristics of the sample are summarized here. These methods are distinct from the analytical approaches that users of the dataset might employ for their own hypothesis-driven research. Descriptive statistics, including means, standard deviations (SD), medians, and ranges, were used to summarize participant demographics, clinical characteristics, and imaging measures. Group comparisons (e.g., cognitively unimpaired vs. impaired) for continuous variables were performed using independent samples t-tests with Cohen’s d for effect size, while categorical variables were modeled using chi-square tests with either the Phi coefficient or Cramer’s V for effect size. To investigate the radiological reads (e.g., Leukoaraiosis, Infarct, Microbleeds) and their association with CDR status, Fisher’s Exact tests, with relevant post-hoc tests, were used on the cross-sectional dataset composed of all last visit variables. Additionally, logistic regressions accounting for age were employed for the evaluation of the association between CDR scores and the presence of microhemorrhages. To examine longitudinal changes in imaging variables (e.g., Cortical Signature, Centiloid, Tauopathy) and their relationship with age and clinical status (CDR), linear mixed-effects models were employed with estimated marginal means calculated for group comparisons. These models accounted for the non-independence of repeated measures within individuals and allowed for the inclusion of covariates as appropriate. The evaluation of WMH volume change used the same approach using log-transformed WMH volumes to approximate a normal distribution. Specific model details, including fixed and random effects, are provided alongside the relevant results in the main manuscript. Additionally, we employed Gaussian mixture modeling to determine PET biomarker cutoffs of amyloid Centiloid and regional tau SUVR.

All statistical analyses for this characterization paper were performed using R statistical software (R Core Team, 2023) and associated packages: *gmodels* for Fisher’s exact tests (Warnes et al., 2018), *rstatix* for Wilcox tests and effect size calculations (Kassambara, 2019), *lme4* for mixed-effects models (Bates et al., 2015), *effectsize* for Cohen’s d calculation (Ben-Shachar et al., 2020), *emmeans* for estimated marginal means and group differences (Lenth, 2022), and *mclust* for Gaussian mixture modeling (Fraley et al., 2012).

### OASIS dataset

Subsets of the KARI dataset have been anonymized and publicly released through the Open Access Series of Imaging Studies (OASIS; https://oasis-brains.org). OASIS provides longitudinal multimodal neuroimaging data with accompanying clinical, cognitive, and biomarker measures from individuals spanning typical aging to those diagnosed with AD. These datasets have supported diverse applications, including hypothesis-driven research, neuroanatomical atlas construction, and image segmentation development. OASIS-1 includes 416 cross-sectional participants aged 18 to 96, 100 of whom over age 60 were diagnosed with very mild to moderate AD (Marcus et al., 2007). OASIS-2 is a longitudinal sample of 150 participants aged 60 to 96, with multiple t1-weighted MRI sessions (Marcus et al., 2010). OASIS-3 extends this effort, comprising 2842 MRI sessions from 1378 participants collected between 2005 and 2020 (LaMontagne et al., 2019). The OASIS-3 Tau dataset includes 451 FTP PET sessions for a subset of participants included in the main OASIS-3 cohort. Importantly, because OASIS represents a static subset of KARI, there is substantial participant overlap; therefore, these datasets should not be combined in analyses to avoid duplication.

### Data Storage and Access

The KARI neuroimaging data, along with associated metadata, are securely stored and managed within the Central Neuroimaging Data Archive (CNDA) (Marcus et al., 2007). The CNDA is built upon the Extensible Neuroimaging Archive Toolkit (XNAT) platform (https://www.xnat.org), a widely adopted open-source informatics platform designed for neuroimaging and related biomedical data. The CNDA provides robust, web-based functionalities for data archiving, searching, visualization, and access, facilitating the management and dissemination of the extensive KARI dataset. The underlying storage infrastructure utilizes a ceph distributed storage system, enabling large-scale clustered data management with built-in redundancy, fault tolerance, and support for disaster recovery. A key feature enhancing reproducibility is the CNDA’s support for Docker container integration; all KARI image processing procedures have been compiled into Docker containers, which helps to ensure that analyses can be replicated across different operating systems and computational environments. This commitment to robust data management and reproducible processing is vital for a shared resource of this scale.

The KARI dataset, encompassing raw DICOM and processed multimodal neuroimaging data, along with associated clinical, cognitive, genetic, and biofluid biomarker information, is made available to the broader scientific research community to accelerate discoveries in AD and related neurodegenerative disorders. Data releases occur biannually, ensuring that the most up-to-date information is accessible. Access to KARI data is managed through the Knight ADRC: Processed volumetric MRI and PET results are generally accessible in tabular formats upon request through the Knight ADRC Biostatistics Core. Source imaging data and derived imaging data can be accessed via secure web download from the CNDA by investigators whose data requests have been approved. Key derived variables are also available in spreadsheet formats directly from the Biostatistics Core. Investigators wishing to access KARI data must submit a formal data request, which includes a description of their proposed research. These requests are reviewed monthly by a KARI faculty committee to ensure appropriate use of the data and alignment with the goals of the Knight ADRC. Requests can be initiated at the Knight ADRC resource request portal: https://knightadrc.wustl.edu/professionals-clinicians/request-center-resources/.

Researchers seeking consultation on specific KARI projects, information on data acquired prior to 2012 (which may have different processing streams), or requiring specialized processing not routinely provided are encouraged to contact the relevant Knight ADRC core leaders directly. Comprehensive documentation, including a detailed data dictionary specific to each data release, is provided to approved users to facilitate their understanding and utilization of the KARI dataset. This documentation can be found at the Knight ADRC resource portal or will be provided with data access. The open yet governed access policy aims to maximize the scientific impact of the KARI dataset while protecting participant confidentiality and ensuring responsible data use.

## Supplementary Material

1

Supplementary Files

This is a list of supplementary files associated with this preprint. Click to download.

- KARIDF25cortsigcentiloidtauopathytssclean.txt
- KARIDF25Cognitionclean.txt
- KARIDF25MRcumulativecountvennclean.txt
- KARIDF25stagingclean.txt
- KARIDF25PETcumulativecountclean.txt
- KARIDF25ClinicalCDRclean.txt
- KARIDF25Demographicsclean.txt
- KARIDF25DemographicsAlluvialclean.txt

## Figures and Tables

**Table 1:** 

Sequence	TotalSessions	Unique Participants			Longitudinal Visits				
			2	3	4	5	6	7	8+
**T1w**	3358	1566	795	457	248	141	80	45	16
**T2-star/SWI**	3000	1495	712	390	210	111	56	20	5
**dMRI**	2641	1394	620	327	170	83	32	12	3
**T2w**	2565	1380	603	318	157	72	26	8	1
**BOLD-rest**	2471	1317	592	313	155	61	21	9	3
**FLAIR**	2016	1110	508	237	100	38	17	5	1
**ASL**	1477	863	339	165	73	25	11	1	0

*Note:* Total counts for each MRI sequence acquired. Total Sessions = total number of individual sessions in which each sequence was acquired. Unique Participants = total number of participants who have at least one session acquired for each sequence. Longitudinal Visits = total number of participants who have at least the given number of acquisitions for each sequence (e.g., 141 participants have 5 or more sessions with a T1w sequence acquired). Average longitudinal lag between sessions was 3.31 years (SD = 1.71).

**Table 2:** 

Tracer	TotalSessions	Unique Participants	Longitudinal Visits				
			2	3	4	5	6+
**Amyloid Summary**	2684	1370	632	335	188	97	48
^**11**^ **C-Pittsburgh Compound B (PiB)**	1848	1108	411	212	83	27	7
^**18**^ **F-Florbetapir (FBP)**	809	660	127	22	1	0	0
^**18**^ **F-Florbetaben (FBB)**	26	17	8	1	0	0	0
^**18**^ **F-Flortaucipir (FTP)**	1113	826	228	55	4	0	0
^**18**^ **F-Fluodeoxyglucose (FDG)**	173	149	24	0	0	0	0

*Note*: Total counts for each PET sequence acquired. Total Sessions = total number of individual sessions in which each tracer was administered. Unique Participants = total number of participants who have at least one PET session for each tracer. Longitudinal Visits = total number of participants who have at least the given number of PET sessions for each tracer (e.g., 188 participants have 4 or more sessions in which any amyloid tracer was administered). Average longitudinal lag between sessions was 3.38 years (SD = 1.56) for amyloid-PET, 3.87 years (SD = 1.55) for tau-PET, and 2.04 years (SD = 1.73) for FDG-PET.

**Table 3:** 

Cross-Sectional	Count	Stable	Count	Increase	Count	Fluctuate	Count	Decrease	Count
0	160	0	960	0 → 0.5	64	0 → 0.5 → 0	12	0.5 → 0	24
0.5	123	0.5	128	0.5 → 1	29	0 → 0.5 → 0 → 0.5	4	1 → 0.5	1
1	62	1	43	0 → 0.5 → 1	10	0 → 0.5 → 0 → 0.5 → 0	3		
2	2	2	1	1 → 2	6	0.5 → 0 → 0.5 → 0	3		
				0 → 1	3	0.5 → 1 → 0.5	2		
				0 → 0.5 → 2	1	0.5 → 0 → 0.5	1		
				0.5 → 1 → 2	1				
**Total**	**347**	**Total**	**1132**	**Total**	**114**	**Total**	**25**	**Total**	**25**

*Note:* Cross-sectional and longitudinal characterization of Clinical Dementia Rating (CDR) scores for the 1643 participants with a neuroimaging session acquired (3T-MRI, amyloid-PET, tau-PET, or FDG-PET), and a CDR assessment within 2 years. Cross-Sectional denotes participants with a single imaging session while Stable indicates longitudinal imaging with no change in CDR. Increase, Fluctuate, and Decrease describe the range of CDR scores obtained in chronological order with repeats removed.

**Table 4:** 

		Totals	Without Impairment CDR = 0	With Impairment CDR > 0	*P*-value	Effect
**Cohort**	Sample Size	1643	1120	523		
**Education**	Mean (SD)	15.86 (2.65)	16.15 (2.46)	15.23 (2.91)	*p* < .0001	−0.35
**Sex**					*p* < .0001	0.11
	Woman (%)	911 (55.45)	663 (59.20)	248 (47.42)		
	Man (%)	732 (44.55)	457 (40.80)	275 (52.58)		
**Handedness**					*p* = 0.87	0.01
	Right (%)	1422 (86.55)	971 (86.70)	451 (86.23)		
	Left (%)	118 (7.18)	78 (6.96)	40 (7.65)		
	Other (%)	103 (6.27)	71 (6.34)	32 (6.12)		
**Race**					*p* = 0.12	0.06
	White (%)	1379 (83.93)	925 (82.59)	454 (86.81)		
	Black (%)	245 (14.91)	179 (15.98)	66 (12.62)		
	Asian (%)	10 (0.61)	8 (0.71)	2 (0.38)		
	Other (%)	9 (0.55)	8 (0.71)	1 (0.19)		
**APOE**					*p* < .0001	0.19
	ε4-positive (%)	691 (42.06)	398 (35.53)	293 (56.02)		
	ε4-negative (%)	952 (57.94)	722 (64.46)	230 (43.98)		

Note: Demographics for the 1643 participants who have at least one neuroimaging session (MRI, amyloid-PET, tau-PET, or FDG-PET) and a Clinical Dementia Rating (CDR) assessment within 2 years. CDR values are the highest value received across study participation. Sex was self-reported by indicating either “Man” or “Woman”. Statistical tests were used to compare distributions of demographic variables between CDR=0 and CDR>0 using either t-test or χ2 tests, with effect sizes showing either Cohen’s d (Age and Education), φ (Sex and APOE), or Cramer’s V (Handedness and Race).

**Table 5:** Biological Staging by Positron Emission Tomography (PET)

Characterization				SessionCount	MeanCentiloid (S/E)	Mean MTL SUVR (S/E)	Mean NEOSUVR (S/E)	MeanTSS (S/E)	MeanTauopathy (S/E)
Stage	Amyloid	Tau - MTL	Tau - NEO						
**AnTn**	**Negative**	**Negative**	**Negative**	529	−2.00 (0.44)	1.09 (0.00)	1.08 (0.00)	0.030 (0.00)	1.17 (0.01)
**A**	**Positive**	**Negative**	**Negative**	144	50.41 (2.02)	1.13 (0.01)	1.10 (0.01)	0.044 (0.00)	1.24 (0.01)
**B**	**Positive**	**Positive**	**Negative**	44	73.58 (3.39)	1.40 (0.02)	1.16 (0.01)	0.125 (0.01)	1.71 (0.03)
**C**	**Positive**	**Positive**	**Moderate**	29	88.23 (5.47)	1.51 (0.03)	1.32 (0.01)	0.304 (0.02)	2.06 (0.05)
**D**	**Positive**	**Positive**	**High**	43	120.88 (6.61)	1.68 (0.03)	1.98 (0.05)	0.683 (0.03)	2.85 (0.08)

Note: Biological staging, counts, and mean PET measures from the 812 instances where a participant obtained a 3T-MRI, amyloid-PET, and tau-PET imaging session all within 1 year. Amyloid-PET Centiloid measures are from mean cortical standardized uptake value ratio (SUVR) with a whole cerebellum reference with a positivity cutoff at 18.42. Tau-PET SUVR measures are from a medial temporal (MTL) ROI with a positivity cutoff of 1.28, and a neocortical (NEO) ROI with a moderate and high cutoff at 1.23 and 1.42 respectively. Cutoffs were determined using gaussian mixture modeling described in supplementary figure 1a, while ROIs are detailed in supplementary figure 1b. An additional 5 sessions were amyloid negative, MTL tau positive, NEO tau negative; 12 sessions were amyloid negative, MTL tau negative, NEO tau positive; 5 sessions were amyloid positive, MTL tau negative, NEO tau positive; and 1 session was amyloid negative, MTL tau positive, and NEO tau positive; possibly reflecting unique AD subtypes or non-AD pathology. AnTn refers to sessions that were amyloid negative and tau negative.
