# Supplementary material for: The Knight Alzheimer Research Imaging (KARI) dataset: a comprehensive multimodal resource for exploring aging, preclinical, and symptomatic Alzheimer disease pathology
Source: Res Sq. 2025 Nov 7:rs.3.rs-7962593. Preprint. [Version 1] doi: 10.21203/rs.3.rs-7962593/v1 (PMC12637817; doi:10.21203/rs.3.rs-7962593/v1)
Supplement: 1 [file NIHPPRS7962593V1-supplement-1.pdf]

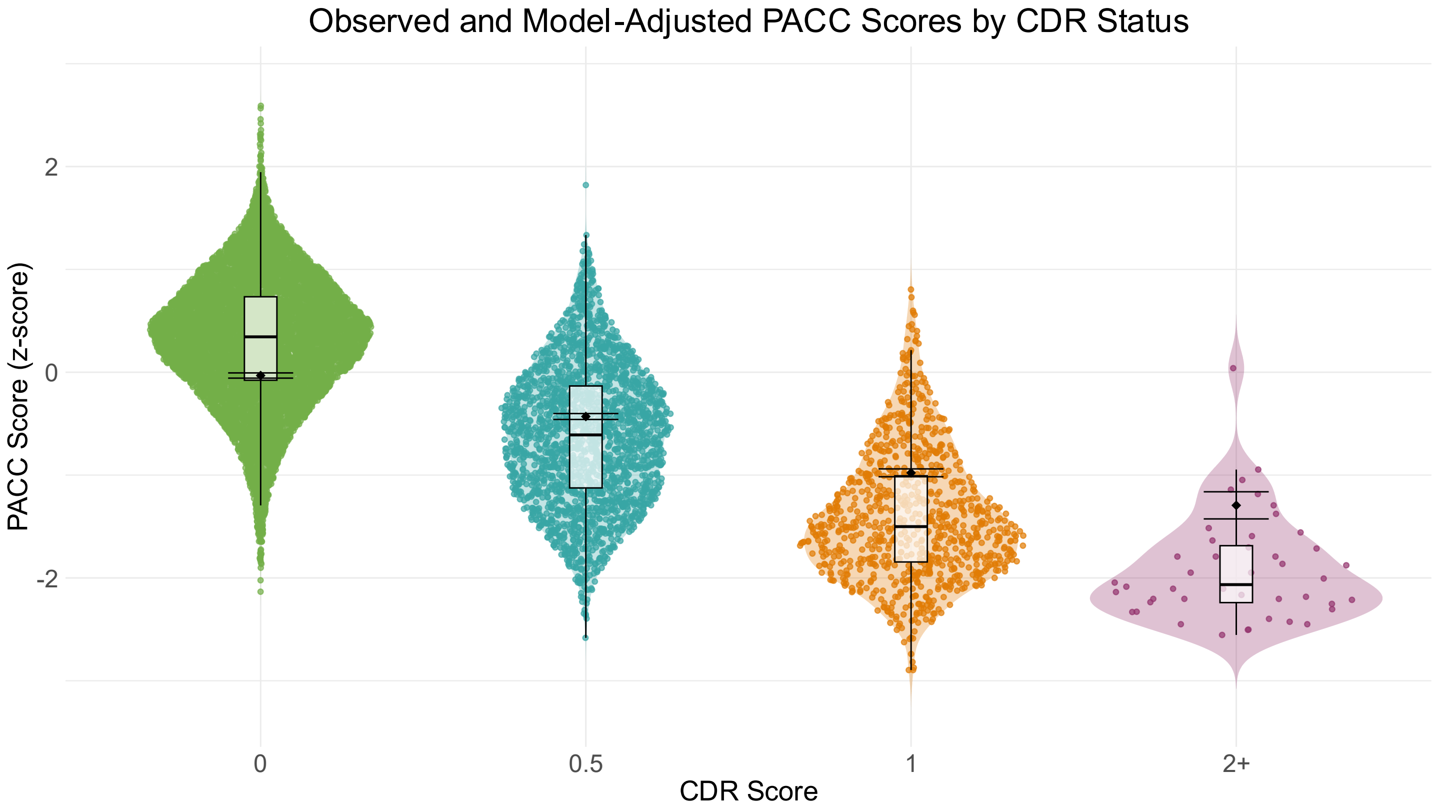

Figure S1

Violin plots depict the full distribution of Preclinical AD Cognitive Composite (PACC) scores (z-scored) for each Clinical Dementia Rating (CDR) group (0, 0.5, 1, 2+). Embedded boxplots indicate the median and interquartile range of observed scores, with whiskers extending to 1.5× the interquartile range. Colored dotes represent individual data points while black diamonds represent estimated marginal means (EMMs) of PACC scores from the linear mixed-effects model accounting for fixed effects of age, education, sex, and APOE ε4 carrier status, and a random intercept for participant to account for repeated measures. Vertical error bars show the 95% confidence intervals around the model-adjusted means. This visualization illustrates both the observed and adjusted patterns of cognitive performance decrease with increasing clinical impairment.

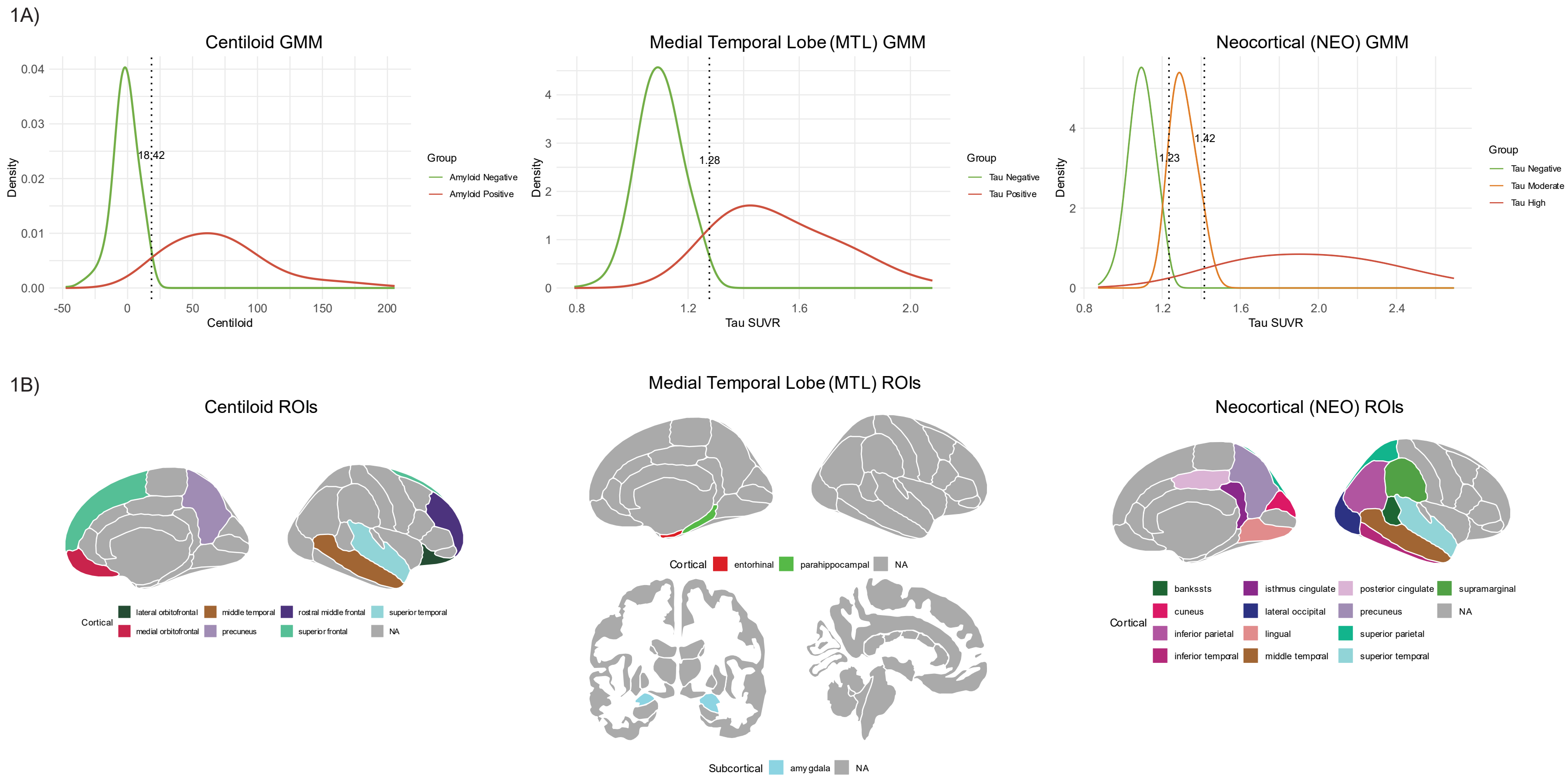

Figure S2

Biological staging methods illustrating A) Gaussian mixture model (GMM) density plots with group cutoffs for Centiloid amyloid-PET, medial temporal Lobe (MTL) tau-PET standardized uptake value ratio (SUVR), and neocortical (NEO) tau-PET SUVR, with a cerebellar cortex reference region and B) Centiloid, MTL and NEO regions of interest (ROI) extracted for PET quantification. Regions derived from the FreeSurfer Desikan-Killiany atlas. Data modeled from the 801 neuroimaging sessions that have a 3T-MRI, amyloid-PET, and tau-PET acquisition within 1 year.
